# Comparison of 16S ribosomal RNA hypervariable regions in microbiome studies of anorexia nervosa

**DOI:** 10.3389/fmicb.2025.1665847

**Published:** 2025-09-26

**Authors:** Arunabh Sharma, Nadia Andrea Andreani, Lara Keller, Beate Herpertz-Dahlmann, Jochen Seitz, John F. Baines, Astrid Dempfle

**Affiliations:** ^1^Institute of Medical Informatics and Statistics, Kiel University, Kiel, Germany; ^2^Max Planck Institute for Evolutionary Biology, Plön, Germany; ^3^Department of Biology and Biotechnology “Charles Darwin”, Sapienza University of Rome, Rome, Italy; ^4^Department of Child and Adolescent Psychiatry, Psychosomatics and Psychotherapy, University Hospital, RWTH Aachen University, Aachen, Germany; ^5^Department of Child and Adolescent Psychiatry, Psychosomatics and Psychotherapy, LVR-University Hospital Essen, University of Duisburg-Essen, Essen, Germany; ^6^Section of Evolutionary Medicine, Institute for Experimental Medicine, Kiel University, Kiel, Germany

**Keywords:** gut microbiome, anorexia nervosa, eating disorder, gut-brain axis, longitudinal study, 16S rRNA, V1V2, V3V4

## Abstract

**Introduction:**

Short read sequencing of the 16S ribosomal RNA (rRNA) gene targeting specific hypervariable regions is widely employed to study the human gut microbiota. In these studies, the selection of particular 16S rRNA hypervariable regions is a crucial step. However, the results of such studies exhibit significant variability depending on the targeted hypervariable region.

**Methods:**

In this study, we systematically evaluated the performance of hypervariable regions V1V2 and V3V4 in a longitudinal gut microbiome study of adolescent patients with anorexia nervosa (AN) and matched controls.

**Results:**

The dominant genera, such as *Bacteroides H*, *Faecalibacterium and Phocaeicola A 858004* were consistently detected in both hypervariable regions across timepoints. The within-sample longitudinal alpha diversity measures varied between the regions with the Chao1 index values being higher in the V1V2 region. The overall microbiome profiles based on beta diversity also differed between the regions. Bland–Altman analysis revealed a general lack of strong agreement between the two sequencing methods, except for a few taxa such as *Faecalibacterium*, *Ruminococcus*, *Roseburia*, *Turicibacter and Anaerotruncus*. While some results were similar across both hypervariable regions, most of the findings were sensitive to the chosen region.

**Conclusion:**

This study underscores the importance of primer selection in microbiome studies of AN, as it can influence taxonomic resolution and diversity estimates along with downstream statistical analyses.

## 1 Introduction

The taxonomic composition of complex microbial communities studied through short-read sequencing of specific hypervariable regions of the 16S rRNA gene has been the standard approach for more than a decade now. A multitude of factors favour the usage of this technique, such as high throughput, previously established computational pipelines with reference databases, and relatively low cost (Fuks et al., 2018; Mukherjee et al., 2018). Such studies employing the 16 s rRNA gene have been able to decipher the varied structure of the microbiota and have greatly contributed to our understanding of the importance of microbiota diversity in human health and disease (Grice et al., 2009; Peciña et al., 2015; Guerrero-Preston et al., 2016; Kehrmann et al., 2017; Mukherjee et al., 2018). This however, comes with a certain caveat: technical variation arising in 16S rRNA amplicon sequencing studies, such as experimental protocols, primer choice, ribosomal hypervariable regions, and selection of reference databases, may considerably influence the identification and classification of microbial taxa (Abellan-Schneyder et al., 2021). For example, previous studies have shown that the choice of amplification primers can influence results by introducing bias (Albertsen et al., 2015; Meisel et al., 2016; Graspeuntner et al., 2018). At the same time, selecting particular 16S rRNA hypervariable regions for sequencing is an important step in 16S rRNA gene sequencing studies. The 16S rRNA comprises nine hypervariable regions, named V1-V9, which are used to identify the taxa and obtain an estimate of evolutionary relationships between them. All nine hypervariable regions give an insight into the microbial taxonomy but the quality and quantity of information that is extracted shows considerable variation depending on the region for a particular studied environment (Heidrich et al., 2022). For example, to study the gut microbiota of Japanese individuals, Kameoka et al. (2021) showed that V1V2 gave more precise estimates for the genus *Akkermansia* than V3V4 based on real-time quantitative polymerase chain reaction (qPCR) assays. Similarly, in a study aiming to characterize the male urinary microbiota, Heidrich et al. (2022) found that V1V2 region provided higher taxonomic resolution when compared to other 16S rRNA hypervariable regions (Kameoka et al., 2021; Heidrich et al., 2022).

Anorexia nervosa (AN) is a severe psychiatric disorder and has a high prevalence in the adolescent age group. Compared to healthy controls, individuals with AN have standardized mortality rates that are 5–10 times higher (Arcelus et al., 2011; Bulik et al., 2019; Andreani et al., 2024). The main characteristics of AN include insufficient energy intake, low body weight, body image distortion, and fear of gaining weight. However, the explanation of the underlying pathophysiology is not well understood (Herpertz-Dahlmann et al., 2021). In recent times, an increasing number of studies have alluded to the role of the gut microbiome in energy extraction from food and weight regulation as well as how it impacts the brain and behavior through the gut-brain axis. Hence, there has been a growing interest in studying the relationship between the microbiome and psychiatric diseases, including AN (Hills et al., 2019; Santacroce et al., 2021; Wu et al., 2021). Additionally, in microbiome studies of AN, a longitudinal design including a post-treatment follow-up, ideally more than 6 months, is advantageous as it helps shed light on the state of the microbiome after weight gain (Andreani et al., 2024) and may help us understand which microbiome alterations may be causes or consequences of the disease.

In AN, 16S rRNA gene sequencing is the most commonly employed method to investigate the role of the microbiome in disease pathogenesis. However, no studies have so far evaluated the implications of selecting a particular hypervariable region in the interpretation of the findings arising from downstream statistical analysis. In this study, we compared the 16S rRNA gene hypervariable regions V1V2 and V3V4 in a gut microbiome study of adolescent patients with AN along with age-matched healthy controls. This work features longitudinal 16S rRNA gene sequencing data, collected during inpatient treatment and at follow-up 1-year after hospital admission.

## 2 Materials and methods

### 2.1 Cohort description and sample collection

The recruitment of study participants and data collection have been previously described (Andreani et al., 2024). Briefly, a total of sixty-four female adolescents (aged between 12 and 20 years, mean 16 years) with AN (typical or atypical) according to the DSM-5 were recruited and enrolled between December 2016 and January 2020 at the RWTH Aachen University Hospital’s Department of Child and Adolescent Psychiatry. Of these, fifty-seven patients were included in the analysis. Stool sampling was done at nine time points, based on prespecified clinical milestones with different interval lengths between patients. These included T0 (admission), T1 (a diet of 25 Kcal/kg/day), T2 (a diet of 50 Kcal/kg/day), T3 (a diet of 62.5 Kcal/kg/day), T4 (a weight gain up to the 5th age-adjusted BMI percentile), T5 (a weight gain up to the 10th age-adjusted BMI percentile), T6 (a weight gain up to the 15th age-adjusted BMI percentile), T7 (discharge), and T8 (1-year follow-up appointment, one year after admission). In addition, 34 age-matched female HCs with normal body weight (between the 20th and 80th percentile of age-adjusted body mass index [BMI-SDS]) were recruited. Faecal sample collection and DNA extraction from stool samples for patients and healthy volunteers have been previously described in detail (Schulz et al., 2021; Andreani et al., 2024).

The study was approved by the ethics committee of RWTH Aachen University Hospital and was carried out in compliance with the Declaration of Helsinki. Before enrollment in the study, written informed consent was obtained from the participants’ parents or legal guardians, and assent was obtained from the study participants themselves.

### 2.2 16S rRNA gene sequencing and processing

The V1V2 region of the 16S rRNA gene was amplified with primers 27F and 338 R using dual barcoding (Caporaso et al., 2011). The resulting library was sequenced on an Illumina Miseq sequencer (250PE). During demultiplexing, no mismatches were allowed in the barcode (Casava, Illumina). QIIME2 (v2019.10) was used to process and analyse the sequence data (Bolyen et al., 2018). Paired end sequences were denoised with ‘dada2’ (Callahan et al., 2016) using default parameters, unless stated: reads were truncated at the first base where the quality score dropped below Q = 3, truncation at 230 bp and the minimum length of reads after truncation was 100 bp.

The V3-V4 region of the 16S rRNA gene was amplified using 515F–806R primers, forward: GTGCCAGCMGCCGCGGTAA and reverse: GGACTACHVGGGTWTCTAAT, with a dual barcoding approach. The resulting library was sequenced on an Illumina Miseq sequencer (300PE). Processing of the reads was performed as previously reported, with the following modification: truncation at 270 bp. An abundance table of 16S rRNA amplicon sequence variants (ASVs) was generated, and the taxonomic annotation of ASVs was obtained using the greengenes2 database (McDonald et al., 2024).

### 2.3 Statistical analysis

All statistical analyses were performed with R (v. 4.4.2). Both datasets were combined into a phyloseq object for all downstream calculations using the phyloseq package (v. 1.46.0) (McMurdie and Holmes, 2013). Using microbial relative abundances, the within-sample alpha diversity (represented by the Shannon and Chao1 indices) was calculated at the genus level and between-sample microbial dissimilarities (beta diversity quantified by Bray-Curtis and Jaccard dissimilarity) were calculated at the genus and ASV levels. The ‘core’ microbiome was defined as those taxa that had a relative abundance greater than 0.01% in more than 50% of the samples. The threshold of 0.01% relative abundance was chosen to ensure that the taxa are present at levels that are likely to have biological significance and not just technical (sequencing) artefacts. This retains the low-abundance taxa and avoids the very rare taxa (near-zero detections). Similarly, the prevalence threshold of 50% maintains that the taxa are sufficiently prevalent and consistently present among the samples. Such *a priori* chosen abundance-occurrence thresholds, although commonly used in microbiome studies, often vary between studies and are study-specific (Neu et al., 2021). We used the R package vegan (v. 2.6.4) (Oksanen et al., 2022) for these calculations. Wherever applicable, the microbial raw counts were transformed into compositional abundances and statistical methods suitable for compositional data were used for analysis. This was done to avoid data loss often associated with rarefaction leading to reduced statistical power (McMurdie and Holmes, 2014). To obtain the summaries of sequencing data, the biomeUtils package (v. 0.022) (Shetty, 2023) was used. Other R packages used for analysis, visualisation and graphics include packages tidyverse (v. 2.0.0) (Wickham et al., 2019), microViz (v. 0.12.1) (Barnett et al., 2021), VennDiagram (v. 1.7.3) (Chen and Boutros, 2011) and ggpubr (v. 0.6.0) (Kassambara, 2023). We used ANCOM-BC2 (Lin and Peddada, 2024) to analyse the microbiota composition for differential abundance testing. We adjusted the *p*-values for multiple testing using the Benjamini–Hochberg (BH) procedure and the taxa that had BH-adjusted *p*-values less than 0.05 were identified as significantly differentially abundant.

#### 2.3.1 Agreement between V1V2 and V3V4 abundance data

The Bland Altman (BA) method (Martin Bland and Altman, 1986) was used as an exploratory analysis to assess the agreement between microbiome relative abundances from the V1V2 (method 1) and V3V4 (method 2) regions for patients at admission. We looked at (a) all common core genera from both methods, (b) alpha diversity indices namely Shannon and Chao1, respectively, and (c) all gut microbial taxa reported in the systematic review by Di Lodovico et al. (2021) that exhibited significant differences in their relative abundances compared to healthy controls. Briefly, in BA analysis, the mean relative abundance and differences were calculated for the methods 1 and 2. Then the mean of the differences (known as ‘bias’) was calculated along with their corresponding standard deviations (SD). Bias quantifies the average difference between the two methods and a low bias is representative of good agreement. Subsequently, the limits of agreement (LOA) were calculated, which provided the range where 95% of the differences are expected to lie. A narrow LOA indicates better precision and less variability between the methods.

We also looked at the agreement between microbiome relative abundances from the V1V2 and V3V4 regions based on the overall microbiome profiles in contrast to BA analysis where individual taxa were considered. For each patient, pairwise Jaccard and Bray-Curtis distances were calculated for the microbiome profiles from both methods to quantify the similarity and dissimilarity between the overall microbiome composition. A Jaccard distance of 0 and 1 represents the maximum similarity (identical microbiome profiles) and dissimilarity (no common microbial taxa) respectively.

## 3 Results

Our cohort consisted of fifty-seven patients who were diagnosed with AN or atypical AN (one patient) according to DSM-5 and aged between 12 and 20 years, as described in detail elsewhere (Andreani et al., 2024). The longitudinal data, including stool samples and clinical data, were collected at nine timepoints, with the first eight (T0-T7) taking place during the treatment at the hospital stay and the last timepoint (T8) at the follow-up appointment 1 year after the hospital admission. Additionally, our study also included a healthy control (HC) group of similar-aged individuals (between 14 and 19 years) whose data were collected at a subset of six time points. A description of the clinical characteristics of the patients and HC groups has been previously detailed (Andreani et al., 2024).

### 3.1 Summary of sequencing data

We compared the sequencing data across the two methods (V1V2 and V3V4) for both the control and the patient groups to gain a better understanding of the characteristics of each method (Supplementary Table S1).

Among patients, the V1V2 dataset had a higher number of total reads (10,575,880) compared to V3V4 (7,164,406) and a higher number of detected taxa (4,593 for V1V2 and 2,104 for V3V4). Meanwhile, V1V2 showed moderately higher sparsity (0.9539098) compared to V3V4 (0.9328799) indicating a greater percentage of undetected taxa among many samples when using V1V2 primers. Similarly, for controls, the V1V2 dataset had a higher number of total reads (3,771,093) in contrast to V3V4 (2,905,102) along with a greater number of detected taxa (3,252) compared to V3V4 (1,518). Like in patients, the V1V2 dataset is more sparse (0.9357342) than V3V4 (0.8964646).

### 3.2 Microbiota composition at important timepoints

**Figure 1:**
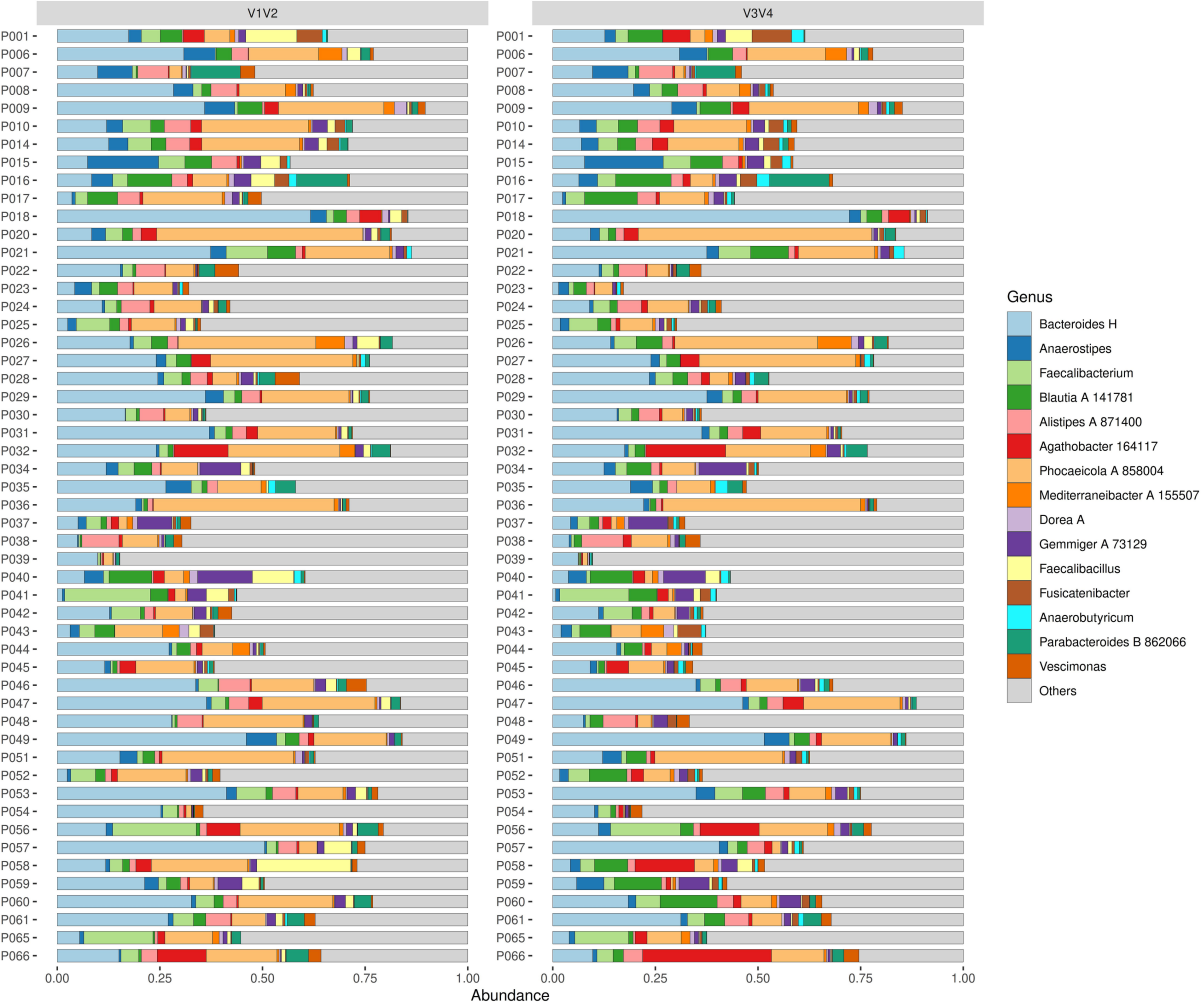
Microbiota composition at T0 in patients with AN. The figure shows the composition of microbes at the genus level for various samples between the V1V2 (left panel) and V3V4 (right panel) sequencing regions. Every sample is represented as a horizontal bar, where the colours describe the relative abundance of the 15 most abundant genera.

To examine the composition of gut microbiome based on relative abundances from regions V1V2 and V3V4 dataset over time, we evaluated taxa composition plots at the genus level for both patients and controls at three different timepoints: admission (T0), discharge (T7), and one-year follow-up (T8) (Figure 1; Supplementary Figure S1).

At T0, all patients showed high relative abundance for genera *Bacteroides H*, *Faecalibacterium*, *Blautia A 141781*, and *Phocaeicola A 858004*. Specifically, *Bacteroides H* and *Phocaeicola A 858004* were predominant in most of the patients and were consistently present in both V1V2 and V3V4 datasets. On the other hand, genera such as *Gemmiger A 73129*, *Alistipes A 871400*, and *Parabacteroides B 862066* showed lower prevalence across both datasets. At T7, the dominant taxa were the same ones from the timepoint T0 for all patients, namely, *Bacteroides H*, *Faecalibacterium*, *Blautia A 141781*, and *Phocaeicola A 858004*. Similar results were also noted for the T8 timepoint, where the microbial community composition showed a prevalence of the dominant taxa across the whole patient group. When we looked at both datasets, in some patients (e.g., P018, P047 and P049), *Bacteroides H* was seen to have higher abundance in the V3V4 region than in the V1V2 region and conversely, *Faecalibacterium* was more abundant in V1V2 than in V3V4 (e.g., P041, P056 and P065). Additionally, both datasets were able to detect low-abundance taxa such as *Parabacteroides B 862066*, *Alistipes A 871400* and *Gemmiger A 73129*. To summarise, the composition of the main genera was relatively consistent across all timepoints in V1V2 and V3V4. Moreover, both datasets showed a similar presence of low-abundance genera, but no region provided definitive evidence to support rare taxa identification.

At T0, genera *Phocaeicola A 858004*, *Bacteroides H*, *Faecalibacterium* and *Gemmiger A 73129* showed a high relative abundance for the control group. Of these, *Phocaeicola A 858004* and *Bacteroides H* were the most dominant genera in most of the control subjects and were consistently present in both the V1V2 and V3V4 datasets. Genera *Faecalibacterium* and *Gemmiger A 73129* were other notable taxa present at T0. We observed a lower abundance of genera *Blautia A 141781*, *Alistipes A 871400*, *Parabacteroides B 862066* and *Roseburia* with no conclusive distinction between the distribution across the two datasets. At T7, the dominant genera from T0 continued to be similarly abundant. Of them, genera such as *Bacteroides H* and *Phocaeicola A 858004* were more noticeable in some individuals compared to T0. Genera *Lachnospira*, *Anaerostipes* and *Anaerobutyricum* showed a slight increase in their detection, while *Dorea A* was seen to be less prevalent but appeared occasionally in some subjects. At T8, most of the taxa that were dominant in the previous timepoints were observed namely *Phocaeicola A 858004*, *Bacteroides H* and *Faecalibacterium* with the addition of *Alistipes A 871400*. Furthermore, the abundance of taxa *Parabacteroides B 862066*, *Blautia A 141781*, and *Lachnospira* were low. Other notable taxa such as *Mediterraneibacter A 155507* and *Fusicatenibacter*, although at lower frequencies, are also seen in certain controls.

### 3.3 Taxonomic overlap

**Figure 2:**
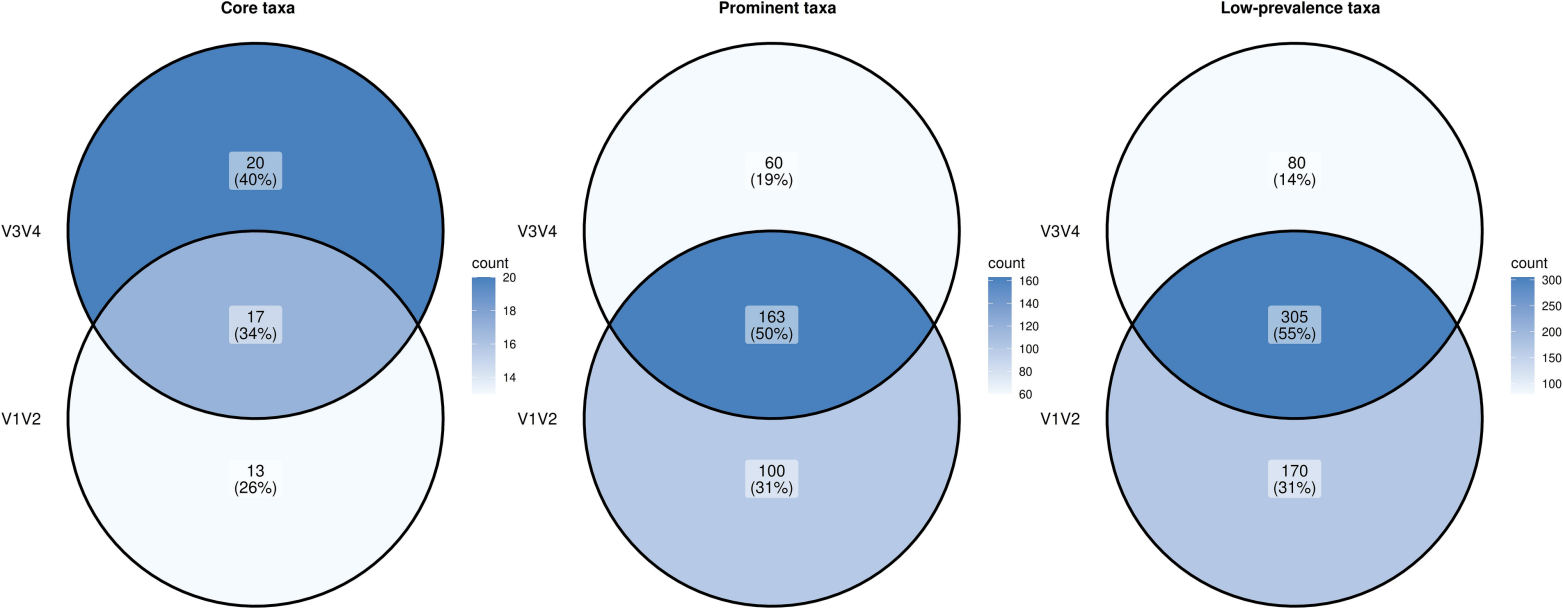
Venn diagram showing the intersection of the core, prominent and low-prevalence microbial taxa between V1V2 and V3V4 datasets.

To understand the overlap and uniqueness of the ‘core taxa’, ‘prominent taxa’ and ‘low-prevalence taxa’ (defined by a prevalence greater than 50, 5 and 0.5% respectively) between V1V2 and V3V4 datasets, among all samples and a detection threshold of 0.01% relative abundance, we looked at their intersection using a Venn diagram (Figure 2). In case of the core taxa, the V1V2 dataset included 13 unique taxa, whereas the V3V4 dataset comprised 20 unique taxa. Seventeen taxa were common to both V1V2 and V3V4, highlighting little overlap (34%) in the core microbiome structure between these two amplicon regions. When we looked at the prominent taxa and low-prevalence taxa, overlaps of 50 and 55% were observed for the taxa derived from the two regions.

### 3.4 Agreement between V1V2 and V3V4 abundance data among patients

The Bland–Altman (BA) analysis was performed to evaluate the agreement between the relative abundances and alpha diversity measures from V1V2 and V3V4 datasets for patients at admission (T0). BA plots visualise the differences in the microbial relative abundances between the two methods against their means.

**Figure 3:**
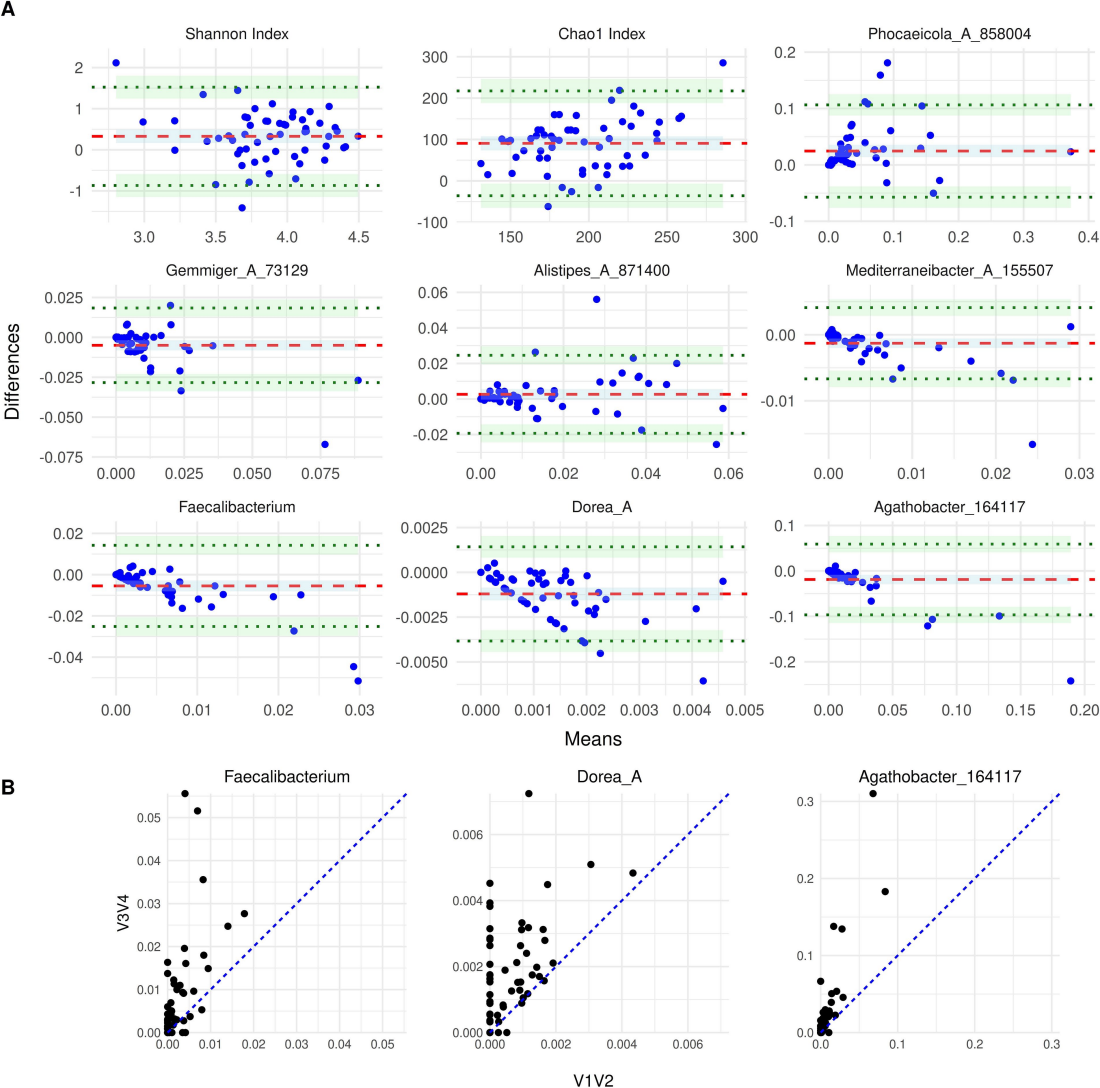
**(A)** Bland–Altman plots of genera at T0. The BA plots compare the taxon abundance between the two sequencing regions: V1V2 and V3V4. The subplots display a specific taxon or alpha diversity measure, where the x-axis denotes the mean abundance of the two methods and the y-axis denotes the difference between the two (V3V4 - V1V2). The red dashed line indicates the mean difference (bias), and the green dotted lines represent the limits of agreement. **(B)** Scatterplot of abundances for *Faecalibacterium*, *Dorea_A* and *Agathobacter_164117* from the V1V2 and V3V4 datasets. The blue identity line (y = x) denotes the ideal case of perfect agreement and deviations from this line reflect differences between the relative abundances from the two regions.

For this analysis, we first looked at a few select dominant genera at admission that formed the common core for both methods, along with a comparison of Shannon and Chao 1 alpha diversity indices (Figure 3A). These genera included *Phocaeicola_A_858,004*, *Gemmiger_A_73,129*, *Alistipes_A_871,400*, *Agathobacter_164117*, *Faecalibacterium*, *Mediterraneibacter_A_155,507* and *Dorea_A*. Of these, *Gemmiger_A_73,129, Agathobacter_164117*, *Faecalibacterium* and *Mediterraneibacter_A_155,507* showed the most consistency between the two methods. They displayed low bias as their mean difference was close to zero, however, with wide limits of agreement (LOA), indicating that there were large absolute differences between abundances calculated from V1V2 data compared to V3V4 data for individual samples. *Alistipes_A_871,400* showed a moderate variability between the methods, although the bias was quite minimal. In contrast, higher variability and systematic trends, indicative of proportional bias, were observed in *Dorea_A* and *Phocaeicola_A_858,004*. In the case of *Dorea_A*, although the overall bias was close to zero, the systematic pattern visible as a decreasing line in the BA plot was due to many patients with an abundance of zero in V1V2 data and abundance values greater than zero in the other dataset (Figure 3B). In the case of *Phocaeicola_A_858,004*, the change in the differences remained systematic across a range of means. An illustration of these differences has been provided in Figure 3B, which shows the scatterplot of abundances in V1V2 vs. V3V4 datasets for *Faecalibacterium*, *Dorea_A* and *Agathobacter_164117*. The BA plot for the Shannon Index indicated consistent overall agreement with tight clustering of points and negligible bias, except for a few outliers. Interestingly, Chao1 Index showed a bias of around 100 with V1V2 dataset consistently overestimating the index relative to the V3V4 dataset.

Then, we systematically looked at the agreement between the two methods for taxa that have previously been associated with AN as listed in Di Lodovico et al. (2021) (Supplementary Figure S2). Among the genera from Di Lodovico et al., genera *Ruminococcus, Roseburia, Turicibacter* and *Anaerotruncus* showed consistent overall agreement between the methods with minimal bias and no apparent bias trends, albeit a few outliers. Genera *Bifidobacterium, Clostridium, Eubacterium* and *Streptococcus*, on the other hand, exhibited systematic trends that suggest proportional biases. For *Streptococcus*, the differences were higher at higher mean abundances and in the case of *Bifidobacterium*, the differences tend to be negative at higher microbial abundances, where the V3V4 dataset yielded consistently higher values. Furthermore, *Anaerostipes* showed a slight funnel-shaped pattern at lower abundances, indicating a slightly increased variability between the methods. The phyla Bacteroidota, Firmicutes and Proteobacteria showed good consistency between methods, indicating negligible systematic differences, although few outliers were observed. Meanwhile, Actinobacteria and Verrucomicrobiota showed negative proportional bias with decreasing differences at higher mean abundances.

**Figure 4:**
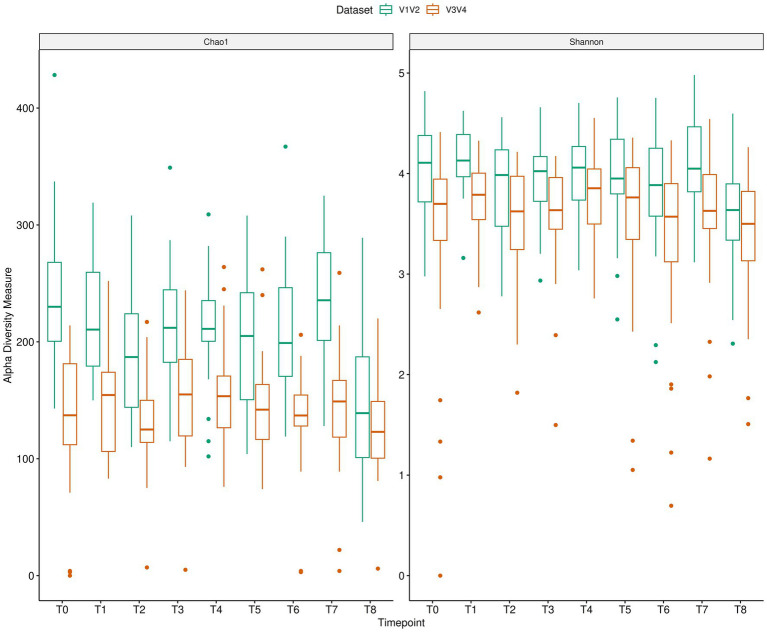
Longitudinal microbiome changes of AN patients depicted using alpha diversity indices (Chao1 and Shannon) for V1V2 and V3V4 data.

We also looked at the relative agreement between V1V2 and V3V4 for alpha diversity indices, Shannon and Chao1 at different timepoints (Figure 4). The BA plots for the Shannon Index (Supplementary Figure S3) at various timepoints did not demonstrate a consistent measurement between V1V2 and V3V4 datasets, although the bias typically neared zero for a majority of the timepoints. Nevertheless, some heteroscedasticity was observed, especially at T8, where differences seem to rise slightly at lower Shannon diversity values. The LOAs were relatively wide, reflecting a weak concordance between the two methods over time. Looking at the Chao1 index (Supplementary Figure S3), the differences between V1V2 and V3V4 data seem to be greater and exhibit more variability than those of the Shannon Index. The average bias remains positively skewed, with the Chao1 index consistently estimating higher richness values. Furthermore, the data points were more dispersed in comparison to the Shannon index. Overall, although both indices showed some degree of consistency between V1V2 and V3V4 datasets, the Chao1 index has greater variability and a more noticeable systematic bias than Shannon.

**Figure 5:**
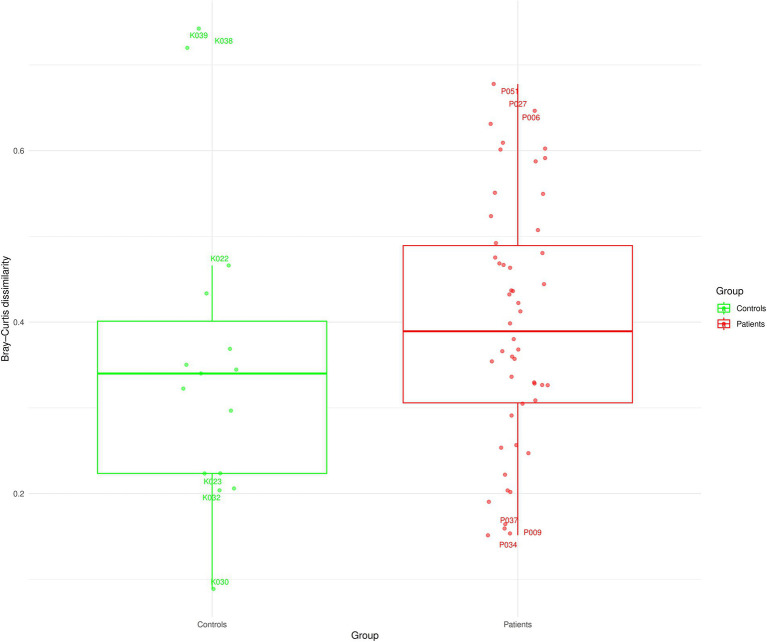
Distance-based analysis based on overall microbiome profiles of patients and controls at the genus level. Each boxplot summarises the within-sample distances (Bray–Curtis) of microbial relative abundances obtained from V1V2 and V3V4 regions, respectively.

Finally, we also compared the agreement between the overall microbiome composition based on relative abundances from both methods for patients and controls. This was based on pairwise Jaccard and Bray–Curtis (BC) distance (beta diversity) for each subject as a measure of overall microbiome profile (dis)-similarity (Figure 5; Supplementary Figure S4). In the patients’ group, the overall microbiome profiles derived from the V1V2 and V3V4 data showed very little similarity between them with a median BC dissimilarity value of 0.38. Interestingly, 20% of the patients had a BC value greater than 0.5, indicating a high degree of dissimilarity between V1V2 and V3V4. In contrast, only 9.2% of patients had a BC value smaller than 0.2, showing good similarity between the methods. Among the patients, P009, P037 and P034 showed the highest similarity; and P051, P027 and P006 are the least similar in terms of their overall microbiome profiles from the two sequencing regions. Similarly, among healthy controls, the two methods were substantially dissimilar (median BC dissimilarity of 0.34). Of these controls, K030, K032 and K023 were the most similar and K039, K038 and K022 showed the highest dissimilarity. Figure 1 and Supplementary Figure S1 show the overall microbiota composition of these patients and controls highlighting the 15 most abundant genera.

### 3.5 Differential abundance analysis

**Figure 6:**
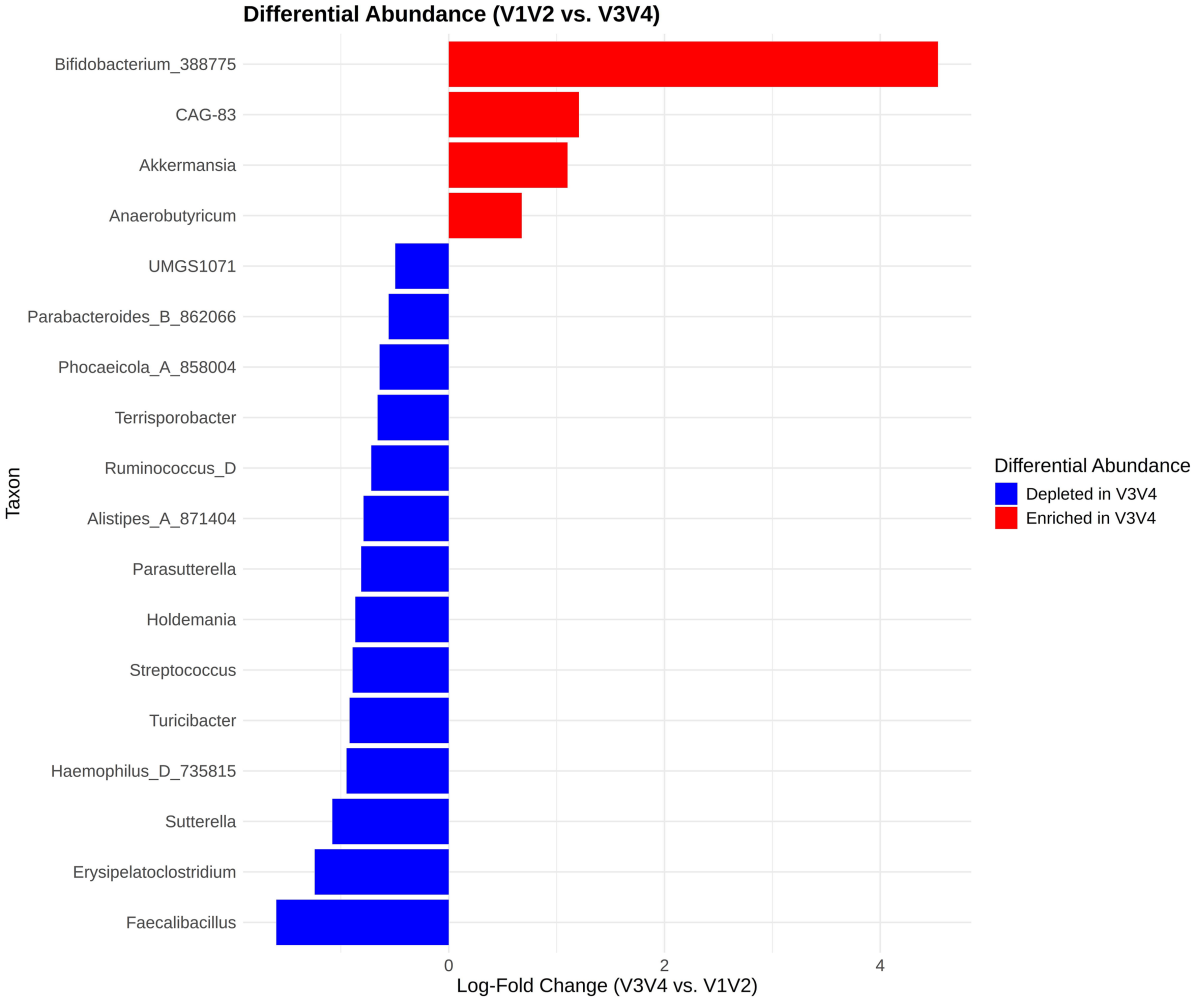
Differential abundance analysis using ANCOMBC2. The *x*-axis displays the log-fold change (LFC) of the V3V4 region relative to the V1V2 region and the *y*-axis shows the list of differentially abundant taxa.

We used the ANCOM-BC2 (Lin and Peddada, 2024) methodology to perform differential abundance analysis to compare the V1V2 and V3V4 datasets at the genus level using all available timepoints for the patient group. A total of 18 genera were identified as significantly differentially abundant after correcting for multiple testing (Figure 6). It is worth noting that since these were the same samples, the differentially abundant taxa reflect the fact that specific taxa were picked up systematically differently by either of the primers used for library preparation. The genera *Bifidobacterium_388775*, *CAG-83*, *Akkermansia*, and *Anaerobutyricum* appeared to be enriched when the V3V4 region was considered. Of these *Bifidobacterium_388775* showed the maximum log fold change, indicating that it is substantially more abundant in the V3V4 region data than in V1V2 data. On the other hand, taxa like *Sutterella*, *Haemophilus_D_735815*, *Turicibacter*, *Faecalibacillus*, *Erysipelatoclostridium* and *Streptococcus* were depleted in V3V4 datasets hinting at the possibility that the V1V2 region might be better able to represent these taxa.

We next performed a BA analysis of the differentially abundant taxa to see how much they differed based on the sequencing region (Supplementary Figure S5). The DA taxa show, expectedly, systematic differences between V1V2 and V3V4 regions as seen in the BA plots. When comparing the two sequencing methods, we see a notable variation in the abundance of these differentially abundant taxa, and in particular, taxa such as *Bifidobacterium_388775*, *Akkermansia*, *Alistipes_A_871404*, *Phocaeicola_A_858,004*, and *Ruminococcus_D*.

## 4 Discussion

Short-read sequencing uses various combinations of primer pairs that target different regions of the bacterial 16S rRNA gene to decipher the composition and abundance of bacterial communities. So far, several studies have compared different combinations of 16S rRNA primers to profile the microbiome in diverse environments (Fadeev et al., 2021; Kameoka et al., 2021; Sirichoat et al., 2021). These studies conclude similarly, arguing that the selection of the 16S rRNA region can significantly impact the analysis of microbiota diversity and composition (Heidrich et al., 2022). Additionally, many previous studies indicate that different regions provide non-concordant outcomes when applied to the same samples (Albertsen et al., 2015; Meisel et al., 2016; Graspeuntner et al., 2018). To our knowledge, these systematic assessments have not been done in the context of AN, resulting in non-consistent and inconclusive literature, due to the variety of methods applied. In this study, we compare the use of 16S rRNA hypervariable regions V1V2 and V3V4 to assess the differences and variability in microbial composition and taxonomic resolution in patients with AN and matched controls using a longitudinal cohort. We systematically look at the agreement of taxonomic abundances between the two regions as it might have an impact on downstream statistical analysis.

We observe that the number of taxa and Chao1 alpha diversity measure detected by amplifying and analysing the V1V2 region is higher compared to the V3V4 region across both patient and control groups. This finding is in line with previous reports, which suggest that the V1V2 region is more sensitive to certain bacterial taxa, capturing more diverse microbes (Heidrich et al., 2022; López-Aladid et al., 2023). Even though the detection of taxa is higher for this region, the region is sparse in terms of proportion. Conversely, the V3V4 dataset shows a higher density despite fewer taxa detected, implying that it could give a more robust identification of common taxa across samples. We also noted a difference in the sequencing depths, where V1V2 yields more reads, which may have resulted in the region detecting a higher number of taxa. This difference is likely attributable to the distinct sequencing chemistries used: V1V2 was sequenced with 250 bp paired-end (250PE) reads, while V3V4 was sequenced with 300 bp paired-end (300PE) reads. The longer read length in the 300PE chemistry typically leads to a greater loss of reads due to reduced quality towards the end of the reads, which may explain the lower read count and subsequently lower number of taxa detected in the V3V4 dataset. These observations and differences reiterate the importance of choosing a methodology and 16S hypervariable region based on the study aims, especially involving the balance of sensitivity and consistency in taxonomic detection.

An important aspect of this study is its design; it looks at both patients and matched healthy controls longitudinally with a focus on three key timepoints for the patients- hospital admission, discharge, and one-year follow-up. We observe that the dominant genera were consistently present across both datasets and sample groups at these different timepoints. Some of the main taxa such as *Bacteroides H*, *Faecalibacterium* and *Phocaeicola A 858004* are detected consistently, suggesting they are present steadily in the gut microbiome over time. However, the low abundant and rare taxa are highly variable and no systematic pattern is observed. This shows that the highly abundant dominant taxa can be expectedly detected across the two sequencing regions but the rare taxa may be more influenced by sequencing depth and biases introduced during the study. An analysis of this study focusing on associations between the microbiome and clinical data was previously published (Andreani et al., 2024).

Furthermore, we used Bland–Altman analysis to compare the agreement of relative abundance measurements between the two sequencing regions to evaluate the biases introduced by the methods. While some taxa, including *Faecalibacterium* and *Mediterraneibacter_A_155,507*, showed sufficient agreement between the methods, most of them exhibited variability and differences. When we looked at alpha diversity at different time points, the Shannon index showed relatively consistent agreement but the Chao1 index indicated variability. We also systematically looked at taxa previously associated with AN (Di Lodovico et al., 2021). Of those AN-related taxa, a few genera such as *Ruminococcus*, *Roseburia*, *Turicibacter* and *Anaerotruncus* showed good overall agreement while the others showed inconsistencies and differences. Taken together, the lack of strong agreement between the methods again illustrates the impact of the choice of the sequencing region can have on profiling the microbiome, especially for certain taxonomic groups.

Our analysis of differential abundance showed that 18 taxa showed significant systematic differences in the same samples when analysed by V1V2 or V3V4 regions. V3V4 dataset showed enrichment of certain taxa, such as *Bifidobacterium_388775*, *CAG-83*, *Akkermansia* and *Anaerobutyricum*. *Bifidobacterium_388775* also exhibited the highest log fold change. In contrast, taxa such as *Sutterella*, *Haemophilus_D_735815*, *Turicibacter* and *Faecalibacillus* were depleted in this dataset. We then performed a BA analysis of these differentially abundant taxa which revealed systematic bias and differences, reiterating that the choice of sequencing region influences abundance estimates and thereby the downstream microbiome analysis and interpretation.

The work discussed here accentuates the significance of methodological considerations in the characterisation of the microbiome of patients diagnosed with AN. At different timepoints, the most abundant genera were frequently observed but it is also important to note that the results from differences in taxonomic detection, diversity estimates, and differential abundance are significant due to potential biases introduced by the choices of selecting the sequencing region. We want to emphasise that when datasets generated using different 16S rRNA regions are compared they need to be interpreted carefully since the sequencing choices affect the resulting taxa detection and relative abundance measurements. Studies should prioritise the sequencing region selection depending on their specific research questions, especially when comparing taxonomic profiles across multiple datasets. In this regard, it is worth mentioning that some studies have tackled these methodological challenges in 16S rRNA-based sequencing. For instance, Fuks et al. have suggested that a combination of two or more hypervariable regions can increase resolution when identifying bacterial taxa (Fuks et al., 2018).

The relative abundance data is intrinsically compositional which could lead to biases while estimating differential abundance. Since our study heavily relies on such data, it could be a crucial drawback and may bias our results. In the future, using absolute quantification methods could provide more robust interpretations of our findings. Furthermore, longitudinal studies including larger sample sizes and metagenomic validation could give us more concrete generalizability of our results. To conclude, we emphasise the importance of choosing an appropriate sequencing region for the study aims as it influences the microbiome profiles significantly. From our study, we observed that the V1V2 region detected a larger number of taxa but indicated higher sparsity as well, while the V3V4 region shows consistency of detection across our samples. Neither of the approaches appears to be systematically better, regardless of these variations. However, the observed differences highlight the variability that is present currently in the literature due to the selection of sequencing regions.

## Data Availability

The datasets presented in this article are not readily available because the informed consent form used for the study does not cover depositing the data in a public repository due to privacy/ethical concerns; since the study participants were minors. Requests to access the datasets should be directed to the corresponding author.

## References

[ref1] Abellan-Schneyder I. Matchado M. S. Reitmeier S. Sommer A. Sewald Z. Baumbach J.. (2021). Primer, pipelines, parameters: issues in 16S rRNA gene sequencing. mSphere 6:e01202-20. doi: 10.1128/mSphere.01202-20, PMID: 33627512 PMC8544895

[ref2] Albertsen M. Karst S. M. Ziegler A. S. Kirkegaard R. H. Nielsen P. H. (2015). Back to basics—the influence of DNA extraction and primer choice on phylogenetic analysis of activated sludge communities. PLoS One 10:e0132783. doi: 10.1371/journal.pone.0132783, PMID: 26182345 PMC4504704

[ref3] Andreani N. A. Sharma A. Dahmen B. Specht H. E. Mannig N. Ruan V.. (2024). Longitudinal analysis of the gut microbiome in adolescent patients with anorexia nervosa: microbiome-related factors associated with clinical outcome. Gut Microbes 16:2304158. doi: 10.1080/19490976.2024.2304158, PMID: 38294867 PMC10832965

[ref4] Arcelus J. Mitchell A. J. Wales J. Nielsen S. (2011). Mortality rates in patients with anorexia nervosa and other eating disorders: a meta-analysis of 36 studies. Arch. Gen. Psychiatry 68, 724–731. doi: 10.1001/archgenpsychiatry.2011.74, PMID: 21727255

[ref5] Barnett D. J. m. Arts I. C. w. Penders J. (2021). microViz: an R package for microbiome data visualization and statistics. J. Open Source Softw. 6:3201. doi: 10.21105/joss.03201

[ref6] Bolyen E. Rideout J. R. Dillon M. R. Bokulich N. A. Abnet C. Al-Ghalith G. A.. (2018). QIIME 2: reproducible, interactive, scalable, and extensible microbiome data science. PeerJ Preprints. doi: 10.7287/peerj.preprints.27295v2 PMC7015180 31341288

[ref7] Bulik C. M. Flatt R. Abbaspour A. Carroll I. (2019). Reconceptualizing anorexia nervosa. Psychiatry Clin. Neurosci. 73, 518–525. doi: 10.1111/pcn.12857, PMID: 31056797 PMC8094122

[ref8] Callahan B. J. McMurdie P. J. Rosen M. J. Han A. W. Johnson A. J. A. Holmes S. P. (2016). DADA2: high-resolution sample inference from Illumina amplicon data. Nat. Methods 13, 581–583. doi: 10.1038/nmeth.3869, PMID: 27214047 PMC4927377

[ref9001] Caporaso J. G. Lauber C. L. Walters W. A. Berg-Lyons D. Lozupone C. A. Turnbaugh P. J.. (2011). Global patterns of 16S rRNA diversity at a depth of millions of sequences per sample. Proceed. National Acad. Sci. 108, 4516–4522. doi: 10.1073/pnas.1000080107, PMID: 20534432 PMC3063599

[ref9] Chen H. Boutros P. C. (2011). VennDiagram: a package for the generation of highly-customizable Venn and Euler diagrams in R. BMC Bioinformatics 12:35. doi: 10.1186/1471-2105-12-35, PMID: 21269502 PMC3041657

[ref10] Di Lodovico L. Mondot S. Doré J. Mack I. Hanachi M. Gorwood P. (2021). Anorexia nervosa and gut microbiota: a systematic review and quantitative synthesis of pooled microbiological data. Prog. Neuro-Psychopharmacol. Biol. Psychiatry 106:110114. doi: 10.1016/j.pnpbp.2020.110114, PMID: 32971217

[ref11] Fadeev E. Cardozo-Mino M. G. Rapp J. Z. Bienhold C. Salter I. Salman-Carvalho V.. (2021). Comparison of two 16S rRNA primers (V3–V4 and V4–V5) for studies of Arctic microbial communities. Front. Microbiol. 12:637526. doi: 10.3389/fmicb.2021.637526, PMID: 33664723 PMC7920977

[ref12] Fuks G. Elgart M. Amir A. Zeisel A. Turnbaugh P. J. Soen Y.. (2018). Combining 16S rRNA gene variable regions enables high-resolution microbial community profiling. Microbiome 6:17. doi: 10.1186/s40168-017-0396-x, PMID: 29373999 PMC5787238

[ref13] Graspeuntner S. Loeper N. Künzel S. Baines J. F. Rupp J. (2018). Selection of validated hypervariable regions is crucial in 16S-based microbiota studies of the female genital tract. Sci. Rep. 8:9678. doi: 10.1038/s41598-018-27757-8, PMID: 29946153 PMC6018735

[ref14] Grice E. A. Kong H. H. Conlan S. Deming C. B. Davis J. Young A. C.. (2009). Topographical and temporal diversity of the human skin microbiome. Science 324, 1190–1192. doi: 10.1126/science.1171700, PMID: 19478181 PMC2805064

[ref15] Guerrero-Preston R. Godoy-Vitorino F. Jedlicka A. Rodríguez-Hilario A. González H. Bondy J.. (2016). 16S rRNA amplicon sequencing identifies microbiota associated with oral cancer, human papilloma virus infection and surgical treatment. Oncotarget 7, 51320–51334. doi: 10.18632/oncotarget.9710, PMID: 27259999 PMC5239478

[ref16] Heidrich V. Inoue L. T. Asprino P. F. Bettoni F. Mariotti A. C. H. Bastos D. A.. (2022). Choice of 16S ribosomal RNA primers impacts male urinary microbiota profiling. Front. Cell. Infect. Microbiol. 12:862338. doi: 10.3389/fcimb.2022.862338, PMID: 35531325 PMC9069555

[ref17] Herpertz-Dahlmann B. Bonin E. Dahmen B. (2021). Can you find the right support for children, adolescents and young adults with anorexia nervosa: access to age-appropriate care systems in various healthcare systems. Eur. Eat. Disord. Rev. 29, 316–328. doi: 10.1002/erv.2825, PMID: 33626222

[ref18] Hills R. Pontefract B. Mishcon H. Black C. Sutton S. Theberge C. (2019). Gut microbiome: profound implications for diet and disease. Nutrients 11:1613. doi: 10.3390/nu11071613, PMID: 31315227 PMC6682904

[ref19] Kameoka S. Motooka D. Watanabe S. Kubo R. Jung N. Midorikawa Y.. (2021). Benchmark of 16S rRNA gene amplicon sequencing using Japanese gut microbiome data from the V1–V2 and V3–V4 primer sets. BMC Genomics 22:527. doi: 10.1186/s12864-021-07746-4, PMID: 34246242 PMC8272389

[ref20] Kassambara A.. (2023). Ggpubr: “ggplot2” based publication ready plots. Available online at: https://cran.r-project.org/web/packages/ggpubr/index.html (Accessed February 26, 2025).

[ref21] Kehrmann J. Veckollari B. Schmidt D. Schildgen O. Schildgen V. Wagner N.. (2017). The lung microbiome in patients with pneumocystosis. BMC Pulm. Med. 17:170. doi: 10.1186/s12890-017-0512-5, PMID: 29202739 PMC5715545

[ref22] Lin H. Peddada S. D. (2024). Multigroup analysis of compositions of microbiomes with covariate adjustments and repeated measures. Nat. Methods 21, 83–91. doi: 10.1038/s41592-023-02092-7, PMID: 38158428 PMC10776411

[ref23] López-Aladid R. Fernández-Barat L. Alcaraz-Serrano V. Bueno-Freire L. Vázquez N. Pastor-Ibáñez R.. (2023). Determining the most accurate 16S rRNA hypervariable region for taxonomic identification from respiratory samples. Sci. Rep. 13:3974. doi: 10.1038/s41598-023-30764-z, PMID: 36894603 PMC9998635

[ref24] Martin Bland J. Altman D. G. (1986). Statistical methods for assessing agreement between two methods of clinical measurement. Lancet 327, 307–310. doi: 10.1016/S0140-6736(86)90837-8 2868172

[ref25] McDonald D. Jiang Y. Balaban M. Cantrell K. Zhu Q. Gonzalez A.. (2024). Greengenes2 unifies microbial data in a single reference tree. Nat. Biotechnol. 42, 715–718. doi: 10.1038/s41587-023-01845-1, PMID: 37500913 PMC10818020

[ref26] McMurdie P. J. Holmes S. (2013). Phyloseq: an R package for reproducible interactive analysis and graphics of microbiome census data. PLoS One 8:e61217. doi: 10.1371/journal.pone.0061217, PMID: 23630581 PMC3632530

[ref27] McMurdie P. J. Holmes S. (2014). Waste not, want not: why rarefying microbiome data is inadmissible. PLoS Comput. Biol. 10:e1003531. doi: 10.1371/journal.pcbi.1003531, PMID: 24699258 PMC3974642

[ref28] Meisel J. S. Hannigan G. D. Tyldsley A. S. SanMiguel A. J. Hodkinson B. P. Zheng Q.. (2016). Skin microbiome surveys are strongly influenced by experimental design. J. Invest. Dermatol. 136, 947–956. doi: 10.1016/j.jid.2016.01.016, PMID: 26829039 PMC4842136

[ref29] Mukherjee C. Beall C. J. Griffen A. L. Leys E. J. (2018). High-resolution ISR amplicon sequencing reveals personalized oral microbiome. Microbiome 6:153. doi: 10.1186/s40168-018-0535-z, PMID: 30185233 PMC6126016

[ref30] Neu A. T. Allen E. E. Roy K. (2021). Defining and quantifying the core microbiome: challenges and prospects. Proc. Natl. Acad. Sci. 118:e2104429118. doi: 10.1073/pnas.2104429118, PMID: 34862327 PMC8713806

[ref31] Oksanen J. Simpson G. L. Blanchet F. G. Kindt R. Legendre P. Minchin P. R.. (2022). Vegan: community ecology package. Available at: https://CRAN.R-project.org/package=vegan (Accessed October 11, 2022).

[ref32] Peciña M. Bohnert A. S. B. Sikora M. Avery E. T. Langenecker S. A. Mickey B. J.. (2015). Association between placebo-activated neural systems and antidepressant responses: neurochemistry of placebo effects in major depression. JAMA Psychiatry 72, 1087–1094. doi: 10.1001/jamapsychiatry.2015.1335, PMID: 26421634 PMC4758856

[ref33] Santacroce L. Man A. Charitos I. A. Haxhirexha K. Topi S. (2021). Current knowledge about the connection between health status and gut microbiota from birth to elderly. A narrative review. Front Biosci 26, 135–148. doi: 10.52586/4930, PMID: 34162042

[ref34] Schulz N. Belheouane M. Dahmen B. Ruan V. A. Specht H. E. Dempfle A.. (2021). Gut microbiota alteration in adolescent anorexia nervosa does not normalize with short-term weight restoration. Int. J. Eat. Disord. 54, 969–980. doi: 10.1002/eat.23435, PMID: 33314258

[ref35] Shetty S. (2023). biomeUtils: utilities for microbiome analytics, p 0. R package version 0.022. Available at: https://rivm-iiv-microbiome.github.io/biomeUtils/index.html

[ref36] Sirichoat A. Sankuntaw N. Engchanil C. Buppasiri P. Faksri K. Namwat W.. (2021). Comparison of different hypervariable regions of 16S rRNA for taxonomic profiling of vaginal microbiota using next-generation sequencing. Arch. Microbiol. 203, 1159–1166. doi: 10.1007/s00203-020-02114-4, PMID: 33221964

[ref37] Wickham H. Averick M. Bryan J. Chang W. McGowan L. D. François R.. (2019). Welcome to the tidyverse. J. Open Source Softw. 4:1686. doi: 10.21105/joss.01686

[ref38] Wu Y. Wang C.-Z. Wan J.-Y. Yao H. Yuan C.-S. (2021). Dissecting the interplay mechanism between epigenetics and gut microbiota: health maintenance and disease prevention. IJMS 22:6933. doi: 10.3390/ijms22136933, PMID: 34203243 PMC8267743

