# Supplementary material for: Comparison of 16S ribosomal RNA hypervariable regions in microbiome studies of anorexia nervosa
Source: Front Microbiol. 2025 Sep 26;16:1665847. doi: 10.3389/fmicb.2025.1665847 (PMC12519842; doi:10.3389/fmicb.2025.1665847)
Supplement: Supplementary file 1 [file Data_Sheet_1.PDF]

# *Supplementary Material*

**Supplementary Table S1:** Comparison of sequencing data across the two methods (V1V2 and V3V4) for both the control and the patient groups.

| | Dataset | Taxa (n) | Sample (n) | Min reads | Max reads | Total reads | Average reads | Singletons | Sparsity |
|---|---|---|---|---|---|---|---|---|---|
| **Controls** | V1V2 | 3,252 | 132 | 1,259 | 71,089 | 3,771,093 | 28,568.89 | 4 | 0.9357342 |
| | V3V4 | 1,518 | 132 | 7 | 45,145 | 2,905,102 | 22,008.35 | 1 | 0.8964646 |
| **Patients** | V1V2 | 4,593 | 299 | 1,618 | 81,769 | 10,575,880 | 35,370.84 | 9 | 0.9539098 |
| | V3V4 | 2,104 | 299 | 0 | 75,983 | 716,4406 | 23,961.22 | 1 | 0.9328799 |

A. Patients at T7

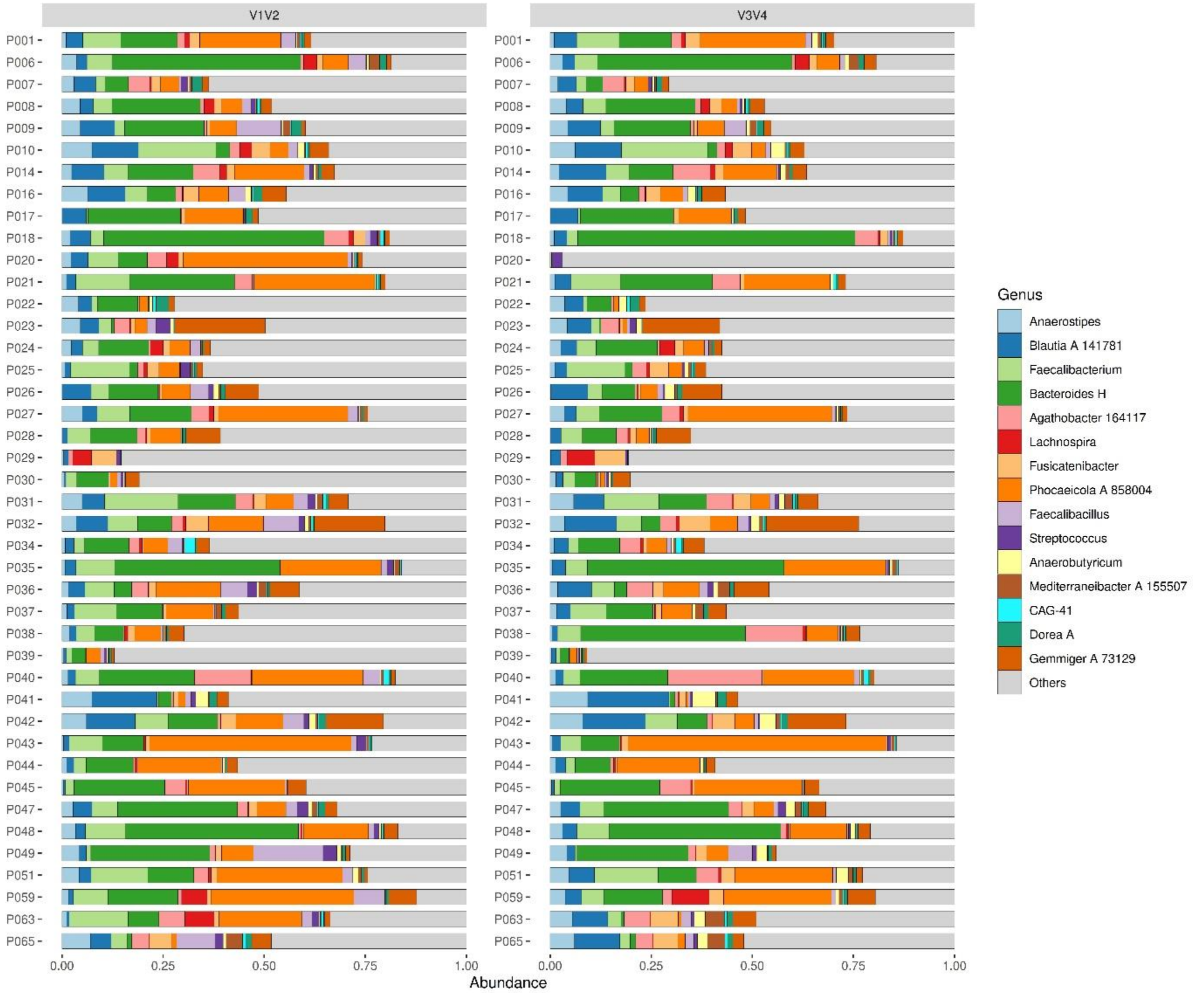

B. Patients at T8

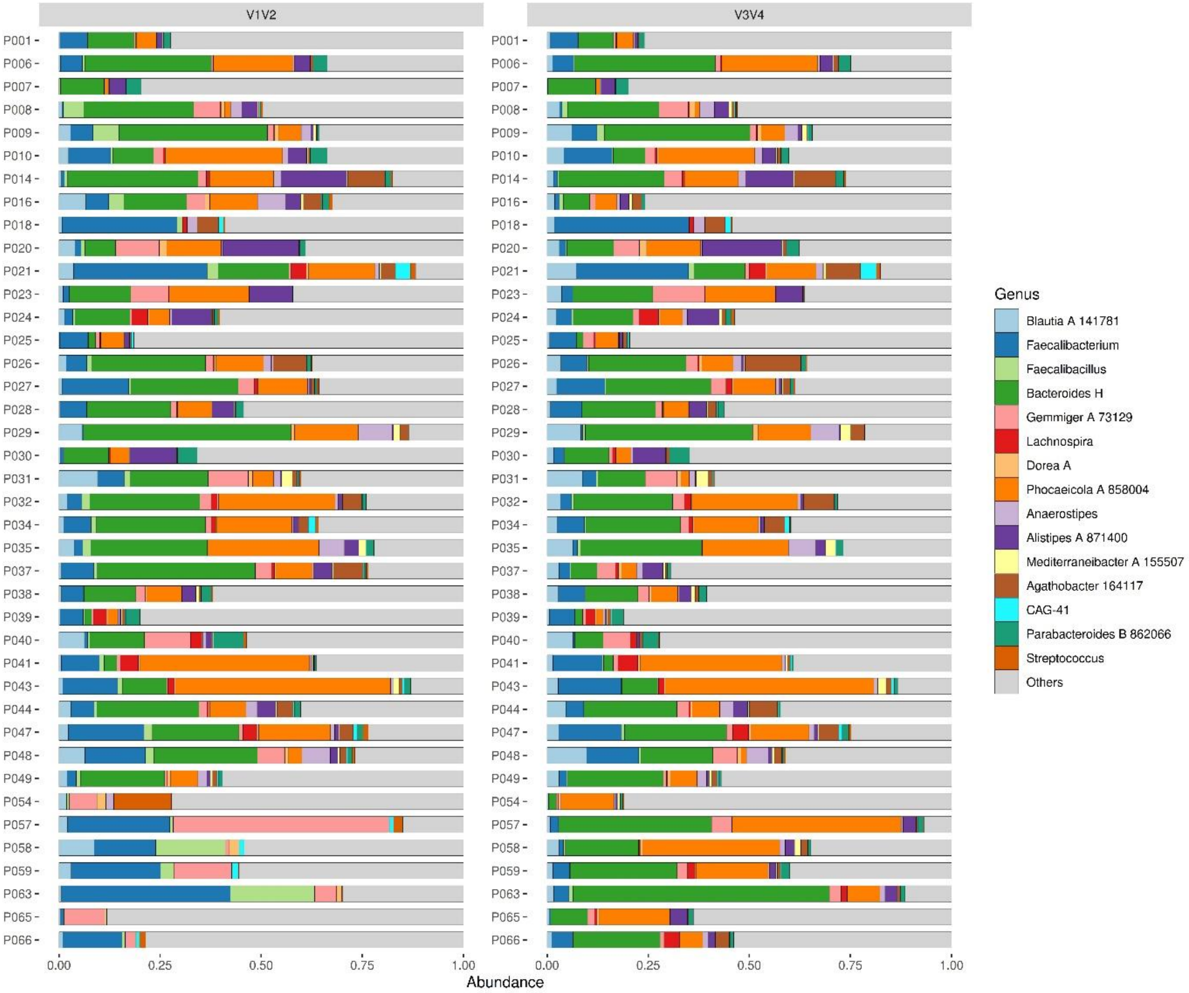

C. Controls at T0

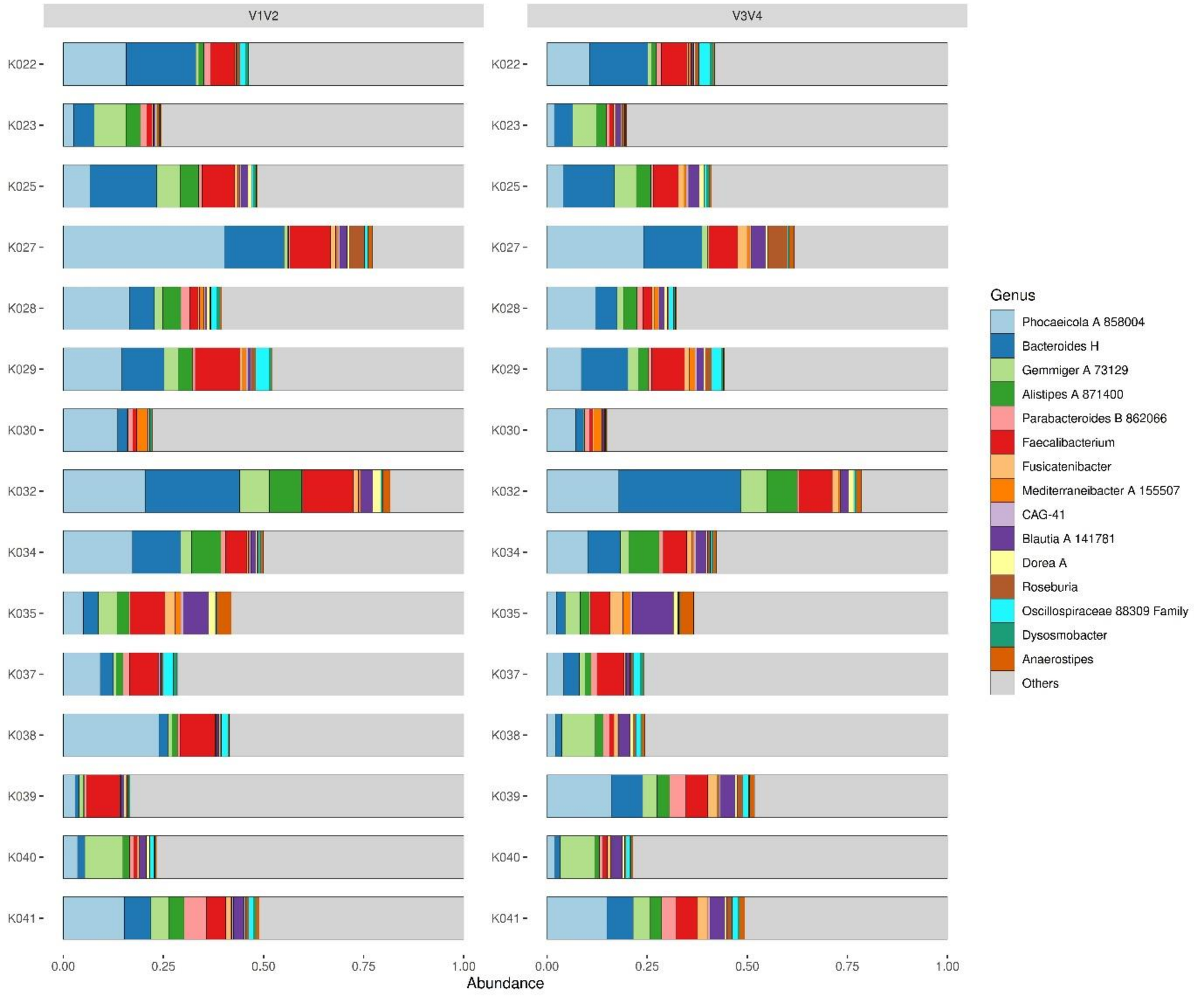

D. Controls at T7

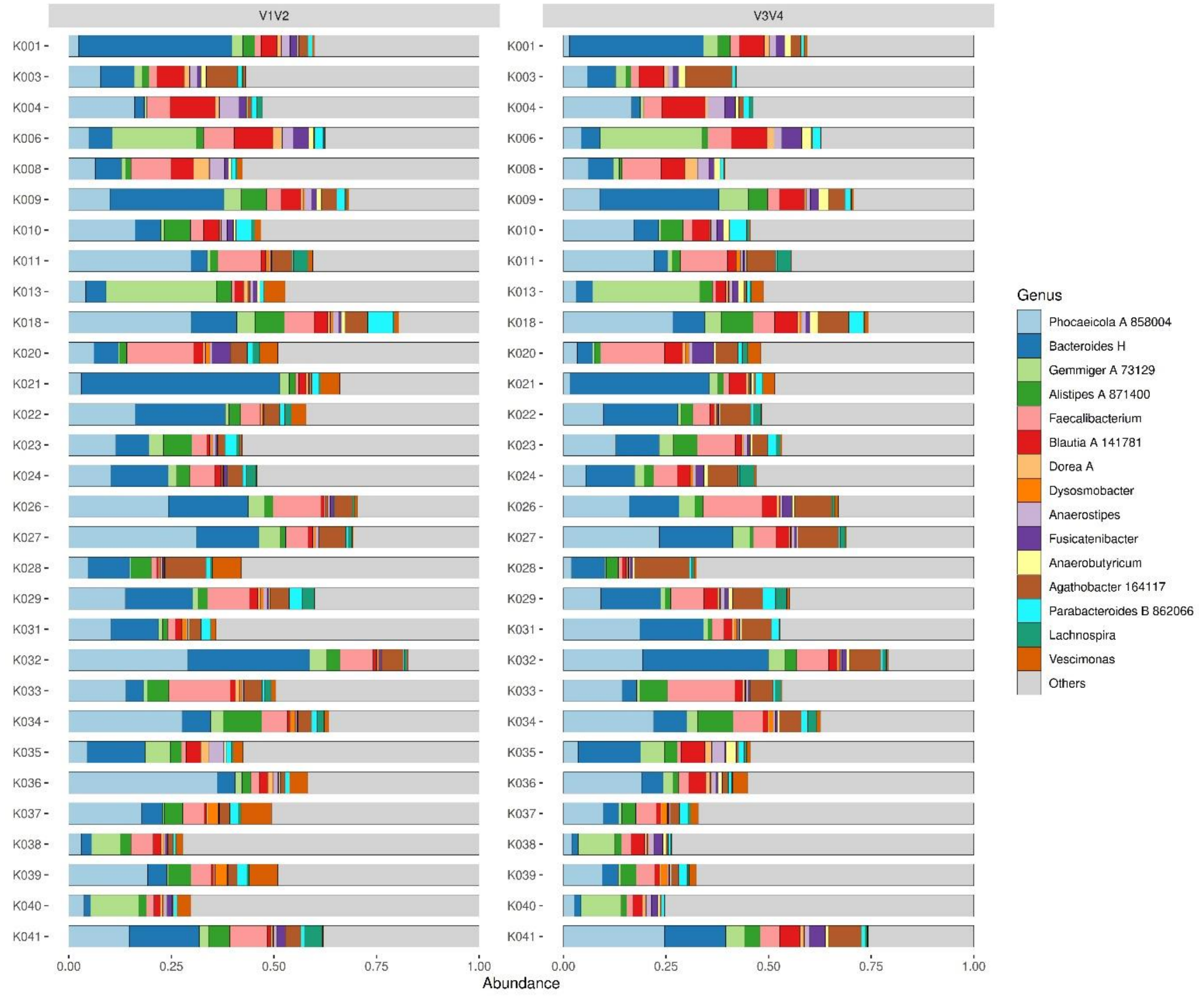

E. Controls at T8

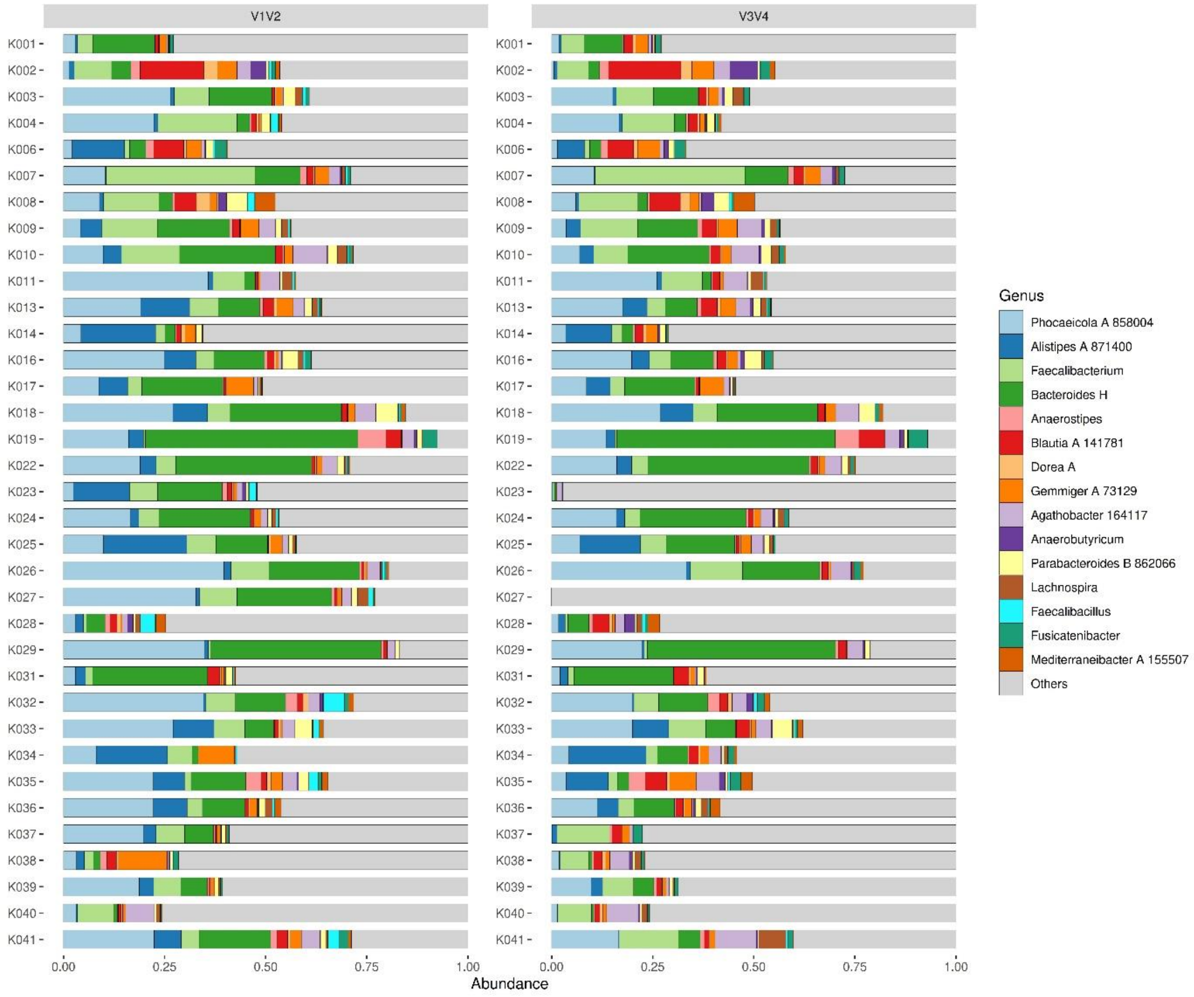


**Supplementary Figure S1 (A-E):** Microbiota composition at T0, T7 and T8 in AN Patients and Controls. The figures show the composition of microbes at the genus level for various samples between the V1V2 (left panel) and V3V4 (right panel) sequencing regions. Every sample is represented as a horizontal bar, where the colours describe the relative abundance of the 15 most abundant genera.

A. BA plots (Genus level)

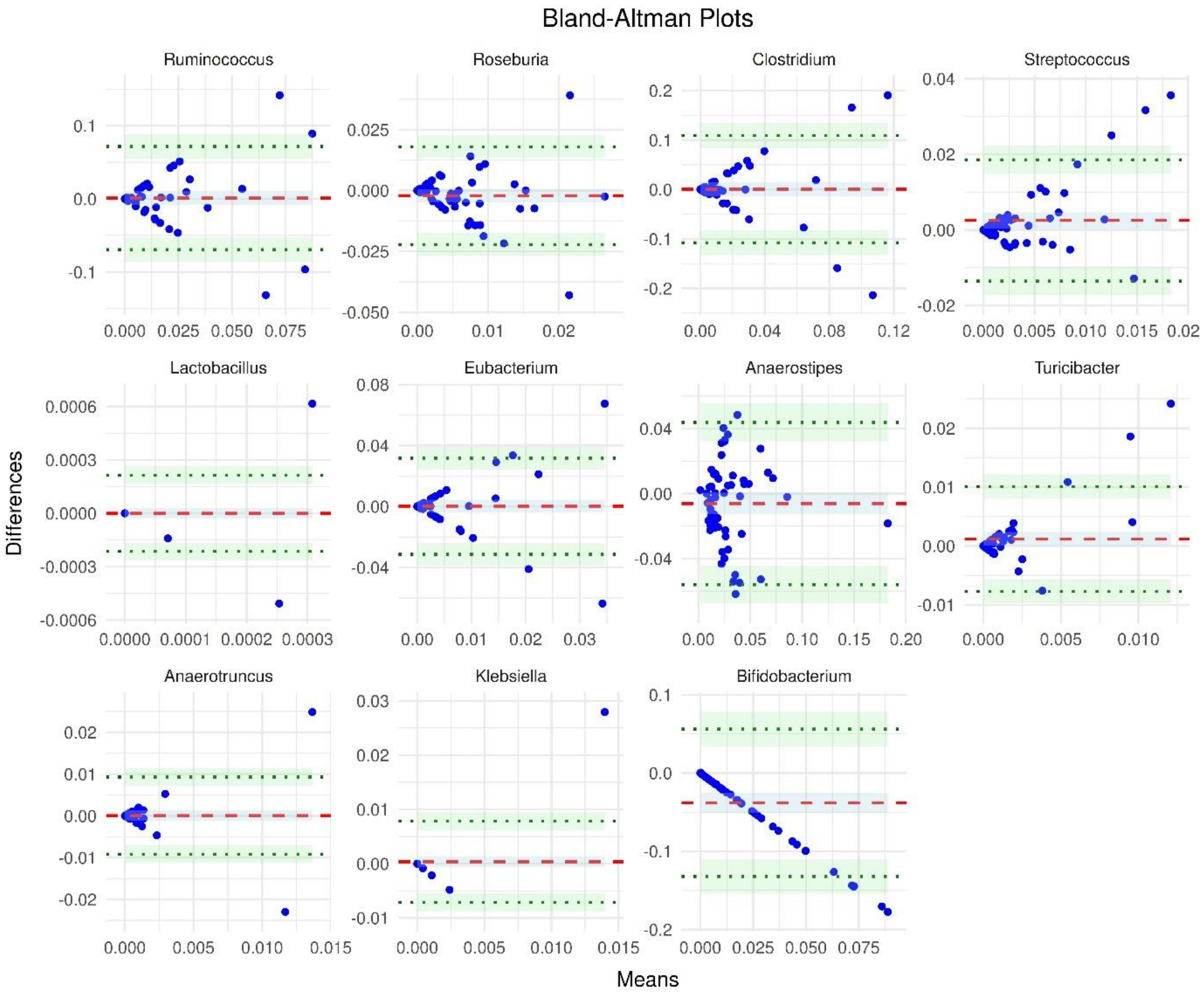

B. BA plots (Family level)

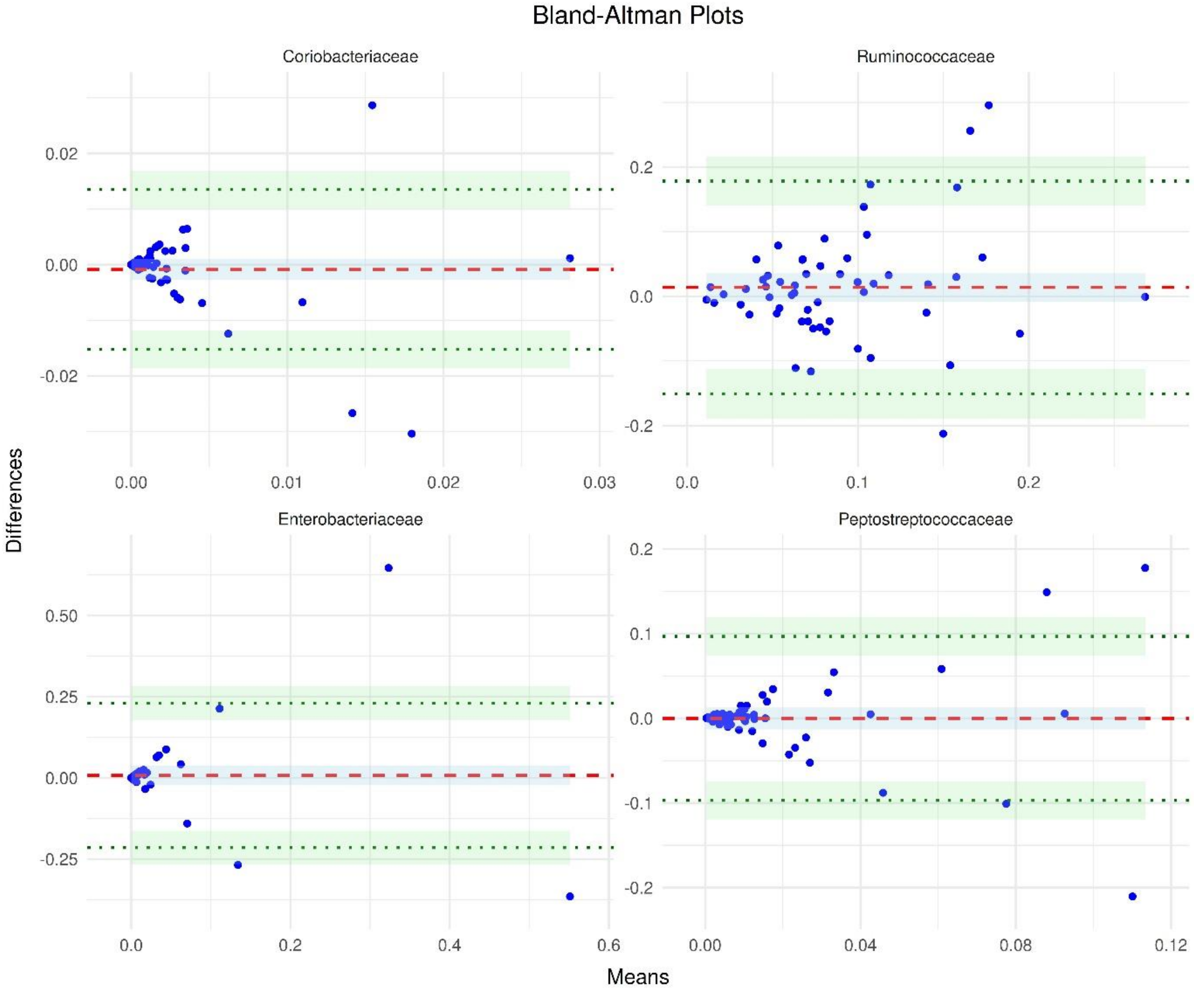

C. BA plots (Phylum level)

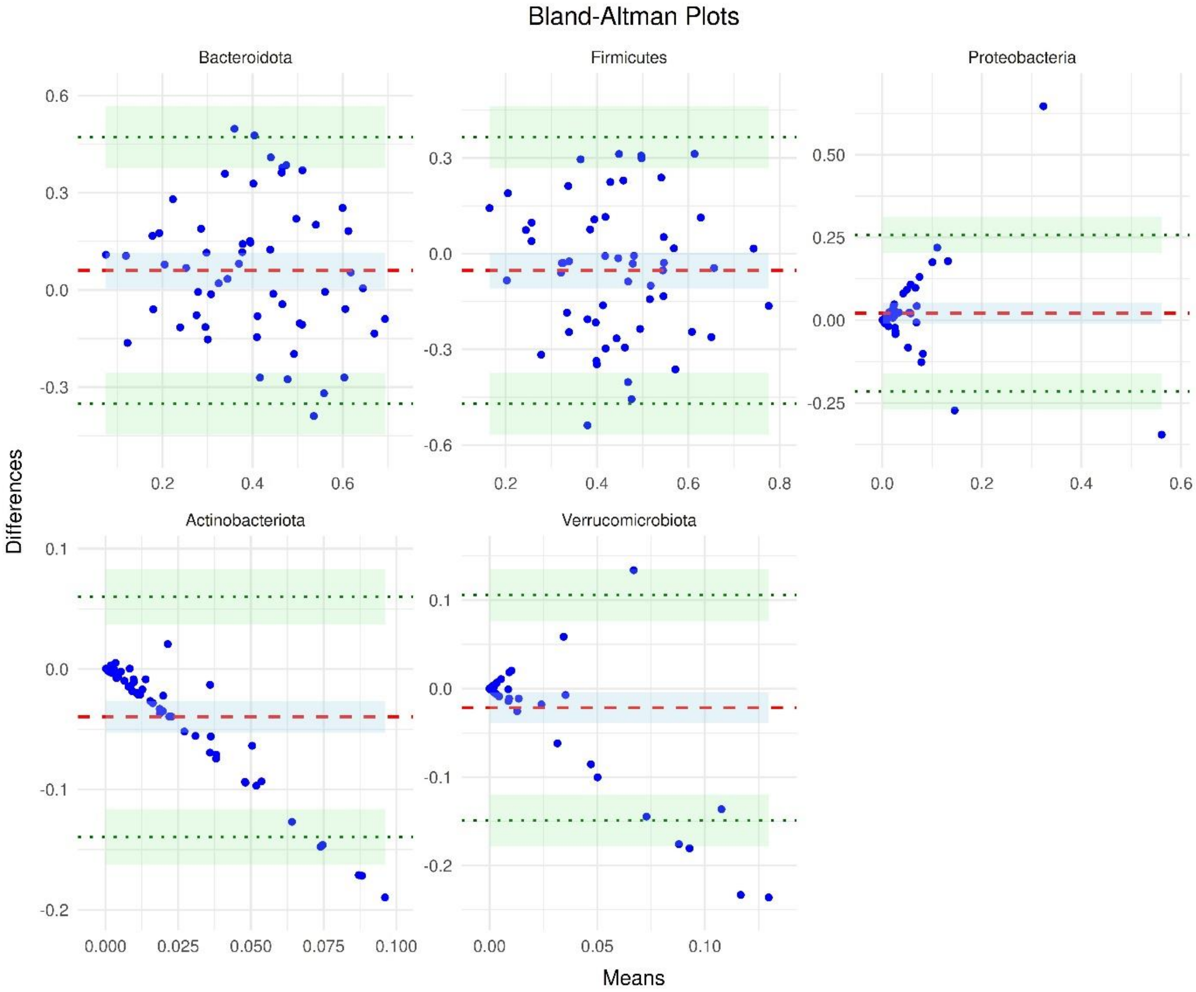


**Supplementary Figure S2 (A-C):** Bland-Altman plots at the genus, family and phylum level reported in Lodovico et al(Di Lodovico et al., 2021). The BA plots compare the taxon abundance between the two sequencing regions: V1V2 and V3V4. The subplots display a specific taxon or alpha diversity measure, where the x-axis denotes the mean abundance of the two methods and the y-axis denotes the difference between the two (V3V4 - V1V2). The red dashed line indicates the mean difference (bias), and the green dotted lines represent the limits of agreement.

A. BA plot (Shannon index)

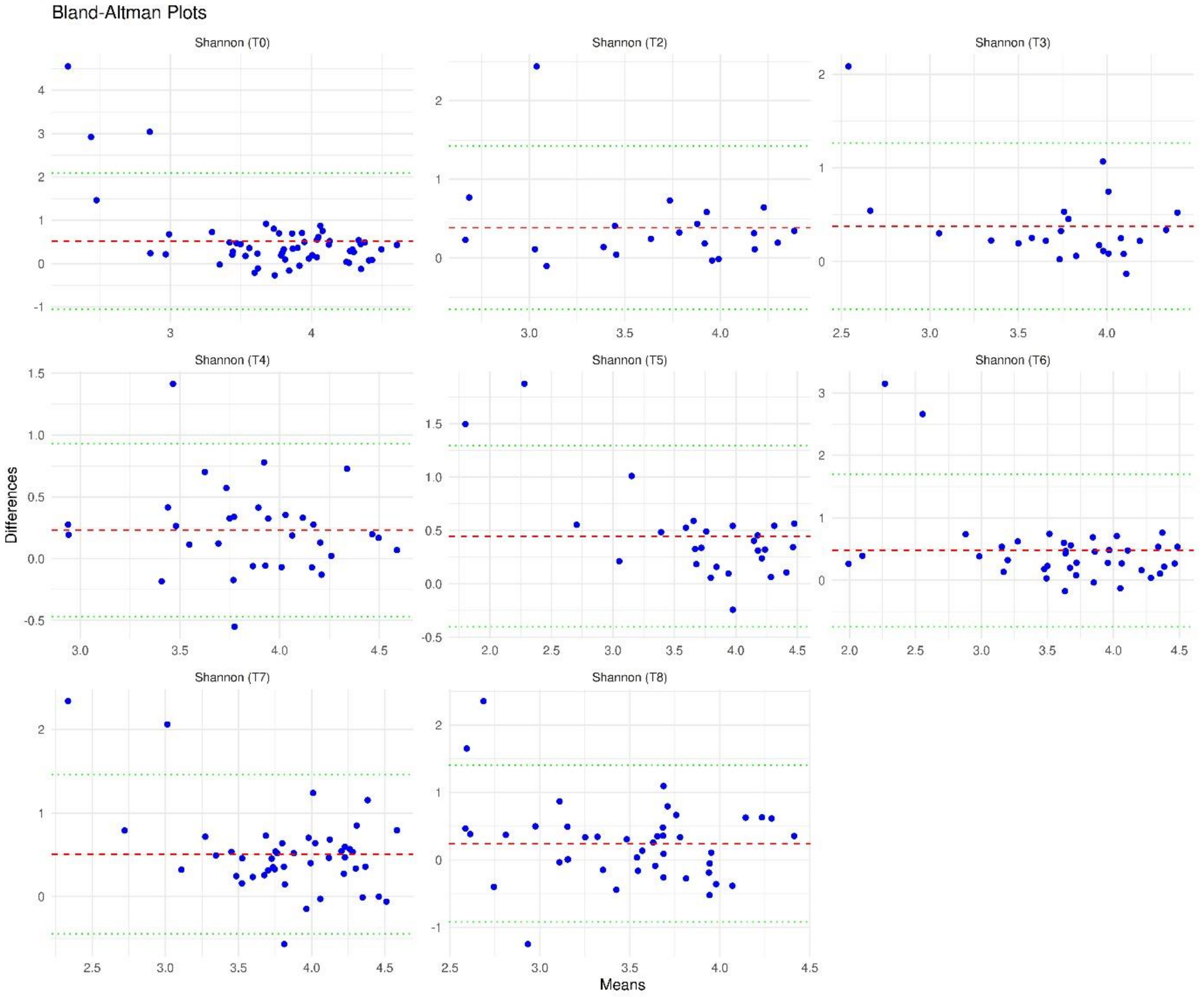

B. BA plot (Chao1 index)

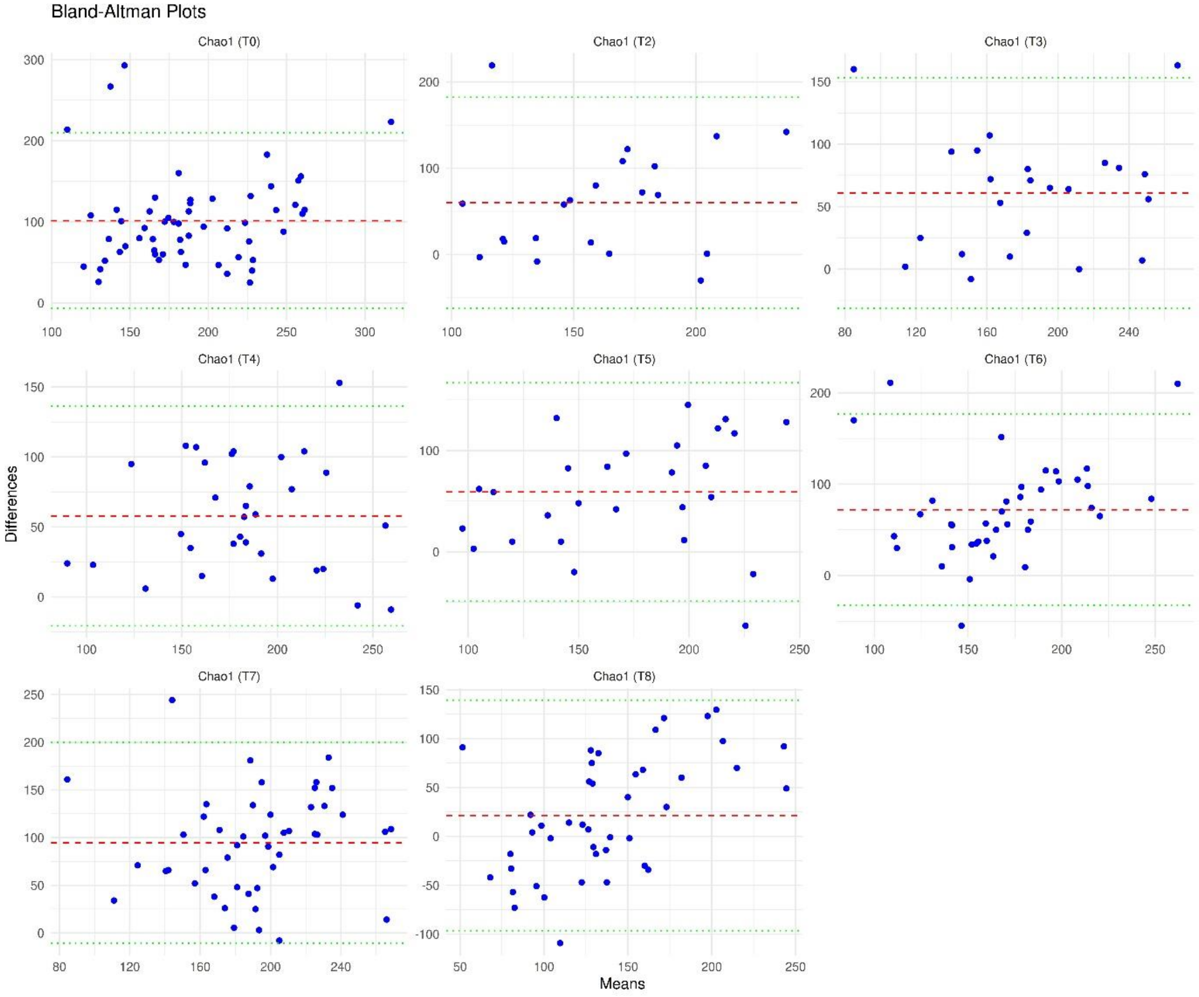


**Supplementary Figure S3 (A-B):** Bland-Altman plots of Shannon and Chao1 indices at various timepoints.

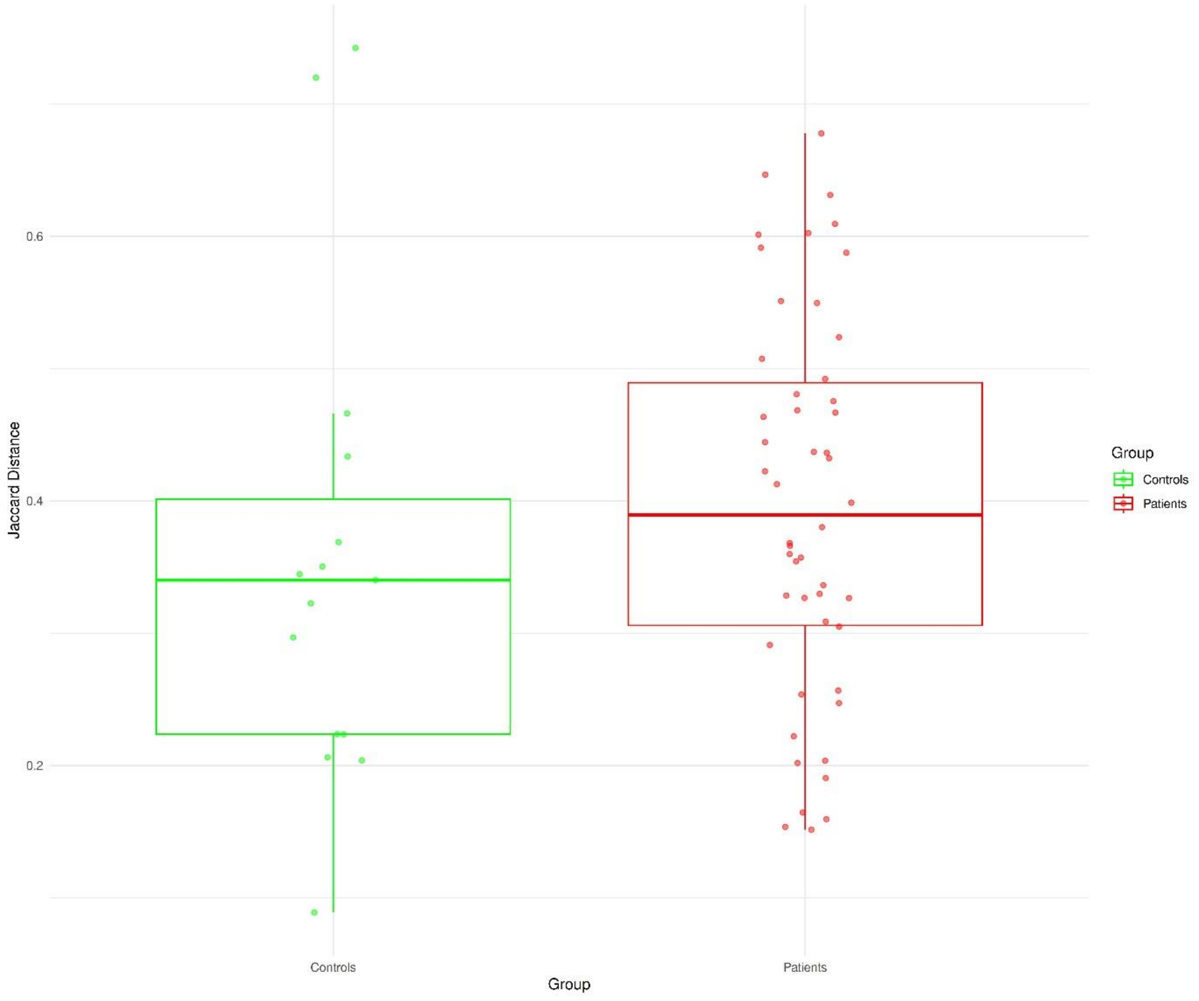


**Supplementary Figure S4**. Distance-based analysis based on overall microbiome profiles of patients and controls at the genus level. Each boxplot summarises the within-sample distances (Jaccard) of microbial relative abundances obtained from V1V2 and V3V4 regions, respectively.

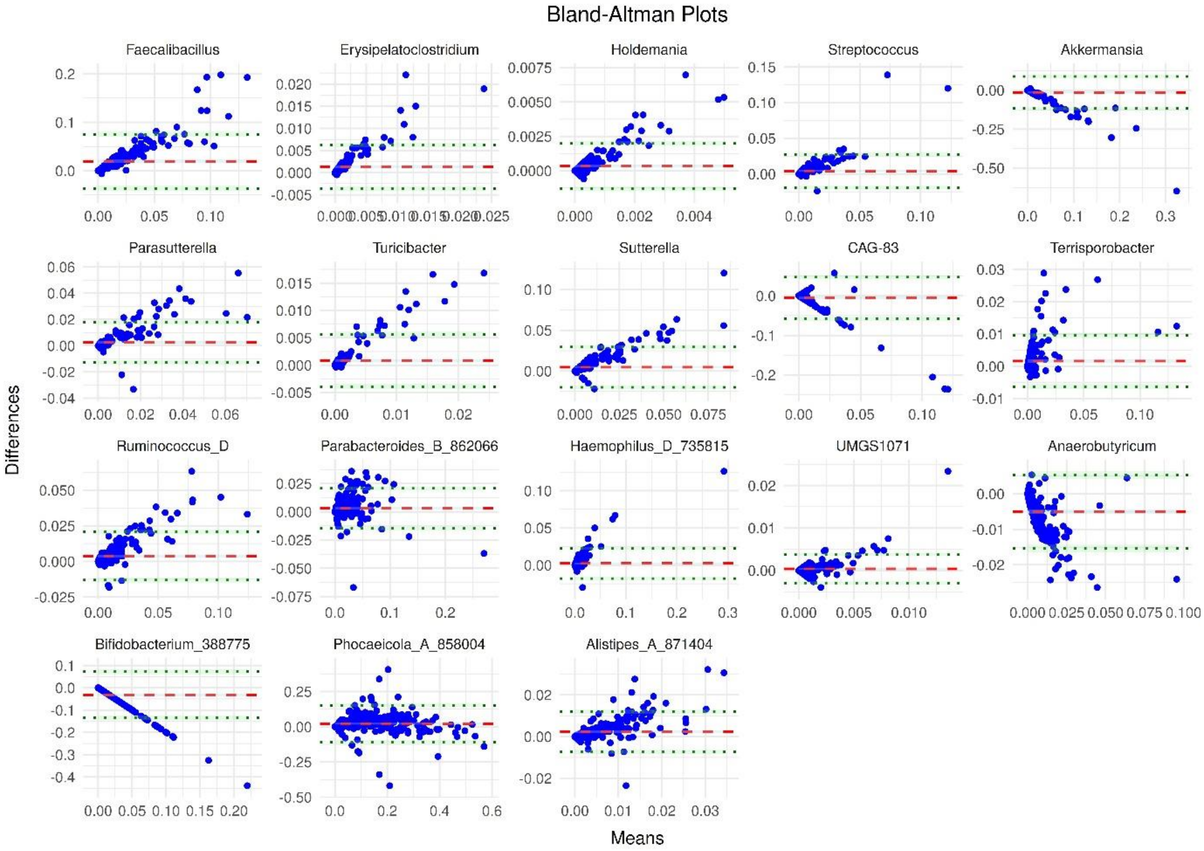


**Supplementary Figure S5**. BA plots of differentially abundant taxa from ANCOMBC2 analysis.
